# “That's All We Wanted, to Be Heard, Listened to, Just to Be Validated and Believed”: Family Experiences of Advocacy Support in Maternity and Neonatal Services

**DOI:** 10.1111/hex.70750

**Published:** 2026-07-03

**Authors:** Rachel Lawrence, Julie Hartley, Nadia Crellin, Nina Hemmings, Holly Walton, Emma Dodsworth, Sarah Fisher, Naomi J. Fulop, Saheli Gandhi, Kevin Herbert, Sonia Macleod, Cate Maddison, Raj Mehta, Stephen Morris, Pei Li Ng, Chris Sherlaw‐Johnson, Ben Wills, Jenny Shand

**Affiliations:** ^1^ Department of Behavioural Science and Health Institute of Epidemiology and Health Care University College London London UK; ^2^ Sands, Pregnancy and Baby Loss Charity UK; ^3^ Research and Policy, Nuffield Trust London UK; ^4^ University College London Public Contributor London UK; ^5^ Department of Public Health and Primary Care University of Cambridge UK; ^6^ Department of Clinical, Educational and Health Psychology University College London London UK

**Keywords:** Independent advocacy service, Maternity care, Neonatal care

## Abstract

**Background:**

Systemic failures in maternity and neonatal care have resulted in adverse outcomes for families. The 2020 Ockenden review (interim findings) recommended creating an Independent Senior Advocate Role as an Immediate and Essential Action. During 2024–March 2026, the Maternity and Neonatal Independent Senior Advocate (MNISA) service was piloted in 16 Integrated Care Boards (ICBs)^1^ and aimed to (1) enable families to feel listened to and heard, with their concerns acted upon, and (2) influence system change. MNISAs supported families following the death or serious injury of a mother or baby during NHS care.

**Objective:**

To explore the experiences of families who did, and did not, receive MNISA support and the perceived impact of MNISAs.

**Design:**

A rapid qualitative study with families who did, and did not, receive MNISA support in 11 ICBs. Interviews were conducted, or written responses to interview questions were collected via an electronic survey tool. All responses were analysed using rapid assessment procedure sheets and inductive thematic analysis.

**Results:**

We interviewed 34 families (*n* = 31 received support and *n* = 3 did not, but were eligible). Findings indicate that MNISAs met service aims by listening to and validating families, enabling their voices to be heard, supporting them to navigate investigative processes and understand their care, facilitating family‐led change. MNISA support was continuous, which helped to alleviate the emotional overwhelm that families experienced. Important barriers to access and engagement were identified. Families who did not receive the service indicated that they would have valued independent advocacy support. All families felt that the service should continue.

**Conclusions:**

Independent advocacy in maternity care creates opportunities for families to be heard and listened to, supporting family‐led change. Further research is essential to understand the ongoing and sustained impact of independent advocacy on family experiences, how the positioning of these services can facilitate equitable access and their role in preventing avoidable harm.

## Patient or Public Contribution

Family members with lived experience of maternity services or adverse outcomes (CM, SF) were involved as members of the core project team throughout the study. We also conducted three Patient, Public, Involvement and Engagement (PPIE) workshops with individuals who had experienced adverse outcomes in maternity care to inform key stages (project development, findings and dissemination). The rapid evaluation team has a wider programme of PPIE leadership (RM, PLN), overseeing PPIE involvement across projects.

## 1 Introduction

The prevalence of adverse outcomes in maternity care has been reported internationally [1, 2, 3, 4]. Evidence shows that despite improvements in the delivery of health care, adverse maternal and neonatal outcomes still occur during pregnancy and birth [1, 2, 4, 5], some of which are preventable [2, 5]. Evidence further highlights disparities in adverse outcomes and experiences of care, attributed to factors such as ethnicity, deprivation and location [3, 6, 7]. The importance of improving maternal health and the delivery of maternity services is therefore a global health priority [2], reflected in the growing number of reviews investigating the quality of care, particularly in the UK [8, 9, 10, 11]. Reviews of NHS maternity and neonatal care in England have identified systemic failings leading to avoidable harm (e.g., stillbirth, neonatal death, serious maternal morbidity or mortality) [8, 9, 12].

Following serious harm or death, families enter a complex landscape of review and investigative processes [5]. Some require prompt decision‐making from families, such as decisions around post‐mortem investigations or consent to medical record disclosure [13]. Internal Trust processes, such as the Patient Safety Incident Response Framework (PSIRF) [14] or the Perinatal Mortality Review Tool (PMRT) [15] (see Appendix 1 for List of Abbreviations), can run alongside, independently or collaboratively with each other. There may also be external independent patient safety reviews (e.g., Maternity and Newborn Safety Investigations (MNSI)) [16], complaint processes or coroner inquests. These reviews and investigations intend to provide families with answers about why their baby or family member died or experienced harm [5, 17]. They also serve a critical purpose for providers by identifying learning to prevent avoidable harm and drive care improvements [5, 16]. These processes can last months or years while families are under immense strain, experiencing the physical, psychological and/or social consequences that can follow serious harm or death [18, 19, 20], and seeking answers, clarity, candour and transparency.

Findings from reviews of maternity care indicate that families can feel silenced or ignored during these processes, and that there is often inadequate learning from serious incidents, with mistakes repeated and limited changes to care [5, 8, 9, 21]. This is despite evidence highlighting the value of involving families in review processes in an open, transparent way, to help them understand what happened and facilitate learning [5, 22]. Access to independent advocacy was first proposed in December 2020 [8], as an Immediate and Essential Action in the interim findings from the Ockenden Shrewsbury and Telford Maternity review. This rationale was reiterated in the final Ockenden report [9], with report recommendations accepted by the Government [23] in March 2022.

Advocacy support for maternity‐related care can be provided through voluntary sector organisations or charities (e.g., Maternity Action) [24], alongside wider health and social care initiatives intended to amplify patient voices and provide feedback to facilitate service improvement (e.g., Healthwatch) [25]. Recent announcements that these services may close have led to concerns about reduced access to independent advocacy for patients [26]. Research from the UK [27] and internationally [28, 29] provides limited evidence on family experiences of *‘advocate’*‐type services in maternity care (e.g., care co‐ordination [28] and non‐clinical community support [27, 29]), suggesting that such services can help to ensure that women and families are listened to, empowered and advocated for throughout their care. However, this research does not report on advocacy roles specifically for families who experience severe adverse maternal and neonatal outcomes. There are few sectors (except some settings like mental health) [30], where independent advocacy is available following harm. The absence of, and variation in advocacy provision, may contribute to the paucity of evidence related to this topic.

The MNISA service was developed and piloted between 2024 and March 2026 to address an Immediate and Essential Action in the Ockenden review [8]. The service aimed to 1) enable women and families to feel listened to and heard, with their concerns acted upon, and 2) influence system change [31]. The pilot was introduced across 16 ICBs in England (Appendix 2). In this context, *‘independent’* refers to independence from the hospitals where harm took place, not from the NHS health care system more broadly. MNISAs provided support to families who had experienced specified adverse maternal or neonatal outcomes during NHS care (Table 1). MNISAs had varied professional backgrounds and experience, ranging from clinical (e.g., midwife and neonatal paramedic) to non‐clinical (advocacy, counselling and patient safety/governance) roles (Appendix 3).

**Table 1:** Adverse maternal and neonatal outcomes that the MNISA service supported.

Stillbirth (after 24 weeks of pregnancy)
Neonatal death
Maternal death
Unplanned or unexpected hysterectomy (within 6 weeks of birth)
Maternal admission to critical/intensive care
A diagnosed or suspected neonatal brain injury (including hypoxic–ischaemic encephalopathy ‐ HIE)

*Note:* There was no time limit on when families needed to experience an adverse outcome to be eligible for the MNISA service [31].

This study formed part of a wider rapid mixed‐methods evaluation of the MNISA pilot conducted between October 2024 and June 2025 by the National Institute for Health and Care Research (NIHR)–funded Rapid Service Evaluation team (RSET) [31, 32, 33], in collaboration with Sands (pregnancy and baby loss charity) [34]. This paper answered the following questions:

1. What were the experiences of families who did, and families who did not, receive support from MNISAs?
2. What impact do families think MNISAs can have?

*‘Experiences’* in this study relate to how families viewed and experienced the MNISA service. The study did not evaluate experiences of clinical care received, i.e., maternity and neonatal care in NHS hospitals.

## 2 Materials and Methods

Ethics and consent statement

All families consented to participate and could withdraw (< 2 weeks after the interview). The evaluation was given favourable opinion by the NHS East of England—Cambridgeshire and Hertfordshire Research Ethics Committee [REC reference: 24/EE/0205] and received Health Research Authority approval. Acknowledging the sensitivity of the research topic, safeguarding protocols were put in place for families, staff and researchers (Appendix 4).

### 2.1 Study Design

A rapid qualitative study was conducted, including online and/or telephone interviews, or written responses to the same interview questions collected via an online survey tool. In line with previous evaluations [35], rapid methods were appropriate, given the pilot time frame, evolving national policy and the need to generate evidence that could help to inform future rollout. Interviews took place between December 2024 and June 2025. The term *‘family/families’* is used throughout to describe parents, partners or wider family members who had experienced an eligible adverse outcome.

### 2.2 Participants and Recruitment

Families were eligible to participate if they had received support from a MNISA or were eligible but did not receive the service. The perspectives of families who did not receive the service were included to explore potential barriers to access, knowledge of the service, and evaluate different views of the service. We aimed to interview up to 40 families (who received support from MNISAs: *n* = 35, and who did not: *n* = 5) across a range of characteristics (e.g., adverse outcome, ICB area, family role – parent or wider relative, demographic characteristics and length of time receiving the service) [31]. Families who received support were recruited by MNISAs and families who had not received support were recruited via online adverts distributed by MNISAs, the Sands's website, maternity and PPIE groups. Open recruitment through social media or other channels (e.g., via hospital Trusts) was not suitable [31] due to the strict eligibility criteria and study sensitivity. Information sheets and consent forms were provided in a range of languages.

### 2.3 Data Collection

Participants provided demographic information verbally or via a secure survey. We offered a variety of ways to participate (online video interview, telephone call and written responses to the interview questions via an online survey tool) [31]. Due to study sensitivity, these different methods were offered to ensure that participation was accessible and that families could contribute in a way that was comfortable for them, an approach consistent with previous research in this area [36]. Interviews lasted approximately 1 hour and were conducted by a researcher from Sands (JH) and RSET (RL/NH/NC). Semi‐structured topic guides were developed (Table 2, Appendix 5). Translated materials and interpretation services were provided where required. Written responses were returned directly into Data Safe Haven (DSH), via RedCap (secure data platform).

**Table 2:** Summary of topic guides.

Interviews with families who had received support from the service	Families who had not received support from the service
Their relationship with their MNISA. How they found out about the MNISA service. The type of support received (with examples). Their experiences of the support received. Any barriers and facilitators to accessing and receiving support from the MNISA service. Whether they think that the MNISA service is meeting the intended aims (with examples). Views on the role specification (e.g., experience and background of their MNISA, independence, etc). Reflections on future practice.	Their awareness of the MNISA service. Reason/s for not accessing the MNISA service. Any barriers and facilitators to accessing the MNISA service (including perceived barriers and facilitators to receiving support from a service like MNISAs). Whether they would have wanted advocacy or other types of support. Whether they think that the service could meet the intended aims. Perceptions on the role specification (e.g., experience and background of their MNISA, independence, etc). Reflections on future practice.

We worked with Sands (JH, BW and SM) and Patient and Public Involvement and Engagement (PPIE) project team members (CM, SF, RM and PLN) to ensure that a comprehensive debrief process was in place (Appendix 4). We co‐developed a national debrief sheet (including national support services, e.g., Sands and Tommy's) (Appendix 6) and specific debrief sheets for different adverse outcomes and local areas. We offered onward referral to the Sands helpline [31].

### 2.4 Data Analysis

All data were analysed in two phases: (i) rapid data analysis and (ii) in‐depth coding. In the first phase, all data were analysed using rapid assessment procedure (RAP) sheets [37] and inductive thematic analysis [31, 38]. RAP sheets enabled data collection and analysis to be conducted in parallel and involved real‐time interview notes being collated into a table, structured around the main interview topics [37]. Two researchers (RL/JH) independently and inductively coded RAP sheet summaries from interviews and written responses (collected via the online survey tool). Researchers then used thematic analysis to develop initial themes. Emerging findings were shared throughout the rapid evaluation [31, 37]. In the second phase, initial themes informed the development of a coding framework. Two researchers (RL/JH) independently applied this coding framework line by line to all transcripts. Quotes were extracted and informed the final themes and subthemes [38]. Researchers (RL/JH/NH/HW/NC) regularly discussed coding and iterative theme development, with input from the wider team. Differences in findings across groups (i.e., received/did not receive the service, different adverse outcomes and family roles) were explored during the analysis. Any key differences are presented in the results. Themes are presented by research question, supported by quotes and tables (see Appendix 7 for additional quotes).

### 2.5 Reflexivity

All team members had different positions and experiences. Some (but not all) researchers had previously accessed, or were accessing, maternity services at the time of the study. PPIE project team members had lived experiences of maternity services or adverse outcomes in maternity and neonatal care. Some researchers were based at universities and others at a pregnancy and baby loss charity (Sands). Discussions during data collection (e.g., after interviews), coding and theme generation helped to manage different positions and any biases.

## 3 Results

### 3.1 Participant Characteristics

**Figure 1:**
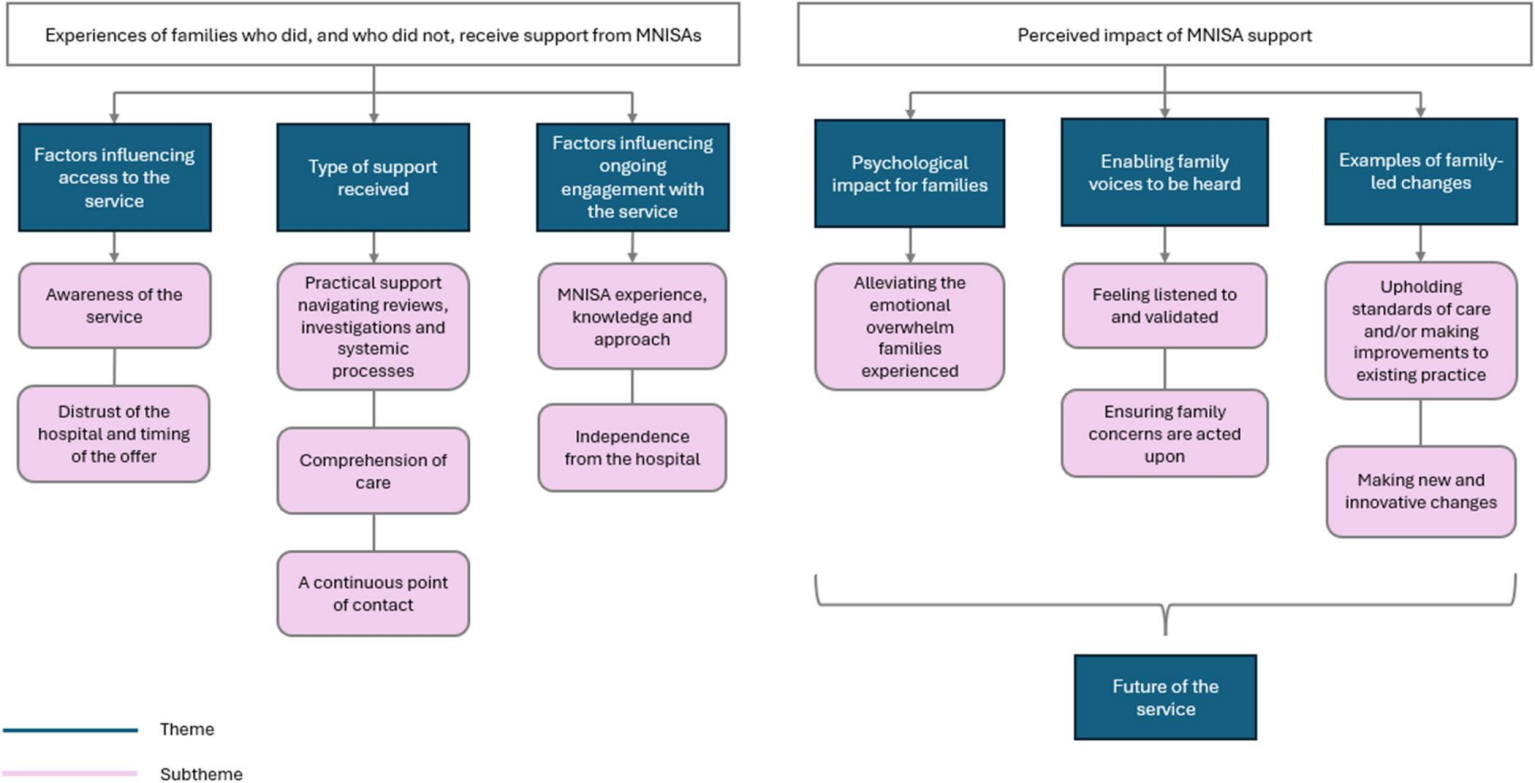
Summary of the study findings.

Thirty‐four families were interviewed across 11 ICBs (Table 3). Interviews were conducted online or via the telephone (*n* = 31), or written responses were provided to the same interview questions (collected via an online survey tool) (*n* = 3). Figure 1 summarises the study findings.

**Table 3:** Participant characteristics.

Demographic characteristics		Families who received the service (*n* = 31)	Families who did not receive the service (*n* = 3)
Integrated care boards *(ICBs represented)*	Number of ICBs	11	2
Adverse outcome *(within the MNISA service)*	Stillbirth	6	1
	Neonatal death	14	1
	Maternal death	2	0
	Unplanned hysterectomy	1	0
	Maternal admission to ICU/critical care	3	1
	Diagnosed or suspected brain injury	9	0
Time since adverse outcome	0–6 months	3	0
	7–12 months	11	0
	1–2 years	9	2
	2–3 years	1	1
	4–5 years	2	0
	Over 5 years	1	0
Sex *(all participants identified with their sex assigned at birth)*	Female	21	3
	Male	11	0
Age	25‐29	7	0
	30‐34	9	0
	35‐39	12	2
	40‐44	3	1
	45‐49	1	0
Index of multiple deprivation (IMD) *(associated with area of residence)*	IMD quintile 1	7	0
	IMD quintile 2	2	1
1 = Most deprived	IMD quintile 3	5	0
5 = Least deprived	IMD quintile 4	7	0
	IMD quintile 5	6	1
Location	Southwest	4	0
	Southeast	5	0
	Midlands	3	2
	London	3	1
	Northeast	11	0
	Northwest	6	0
Ethnicity *(self‐reported)*	White British	25	2
	British Pakistani	1	0
	British Indian	1	0
	Indian	1	0
	White Romanian	1	0
	White Albanian	1	0
	Asian Pakistani	2	0
	White European	0	1
Primary language	English	27	3
	Punjabi	1	0
	Albanian	1	0
	Romanian	1	0
Disability or long‐term condition	Yes	3	0
	No	27	3
Referral pathway to the MNISA service	Given a leaflet (from a midwife and/or hospital staff)	4	
	Investigation team	4	
	Bereavement midwife	4	
	Counselling team	2	
	Perinatal mortality review team	2	
	Reproductive trauma team	2	
	Head of local neonatal unit	1	
	Perinatal mental health team	1	
	Hospital staff (role not specified)	1	
	Patient Advice and Liaison Service (PALS)	1	
	Hospice charity	1	
	Friend who worked in the NHS	1	
	Head of midwifery	1	
	Maternity and Neonatal Voices Partnership Facebook page	1	
	BBC news	1	
Reason for not accessing the MNISA service	Did not know about the service/were not told		2
	Did not know about the service and found out too late – not the right time for them and complex context with national reviews ongoing		1

*Note:* Figures for some demographics will not always match the total sample size due to, for example, two participants in one interview, one family experiencing multiple adverse outcomes and multiple routes of referral/access or families choosing not to share demographic details (either overall or just for certain characteristics).

All the families we spoke to were having at least one, but usually more than one, investigation process to help determine if the care the mother or baby received from the hospital contributed to their severe injury or death. These include PMRT, PSIIs, PSIRF, MNSI, coroner's investigations, coroner's inquest, Child Death Reviews and complaints (see Appendix 1 for List of Abbreviations).

## 4 Experiences of Families Who Did, and Who Did Not, Receive Support From MNISAs

### 4.1 Factors Influencing Access to the Service

#### 4.1.1 Awareness of the Service

Families were initially hesitant about accessing the MNISA service, and this finding was evident across the range of adverse outcomes that MNISAs supported. Those who did not receive the service were unaware of MNISAs, or they found out too late to benefit from advocacy support (Table 3). Many described feeling unsure as to why they were not told about MNISAs and how independent advocacy could have helped them. Families accessing the service also lacked clarity about how MNISAs could help them, with trust, rapport and understanding of the role only developing after they met with their advocate. This first meeting was when their advocate explained their role (e.g., being their independent voice, helping them navigate investigative processes and feedback to the hospital):

“Because she [perinatal mental health co‐ordinator] didn't want to leave me with no one, she passed me onto [my MNISA]. I thought how is [my MNISA] going to help me, what's she going to do for me? Then I met her and we clicked instantly, I saw how she can help a lot with the practical side of the investigation. [When my MNISA] came along, I realised how much we needed her”.

(Mother whose baby died in the neonatal period, INT1^1^)

#### 4.1.2 Distrust in the Hospital and Timing of Offer

Additional barriers included the perception that the MNISA represented yet another person in an already complex landscape—someone who might let them down when trust had been broken and make processes harder rather than easier to manage. The offer of support also often came at a time when families were navigating not only their grief but their distrust of the hospital:

“I don't recall what [the hospital] said about it, they just mentioned it. I remember my first thought being, ‘This going to be another person that's hospital‐based, another person from the NHS, that's going to get involved and muddy the waters’. I remember thinking, do we need some sort of advocate? We've got this investigation that's about to start, have we got the mental capacity to deal with somebody else? As soon as we met, she explained everything, who she was and what she did. I got the picture then and thankfully we said, ‘Yeah we'll speak to this advocate’. Once it's explained it's an absolute no brainer”.

(Father whose baby died in the neonatal period, INT27)

### 4.2 Type of Support Received

#### 4.2.1 Practical Support Navigating Reviews, Investigations and Systemic Processes

The reviews and investigations that are meant to help families can also be very difficult and overwhelming to navigate. Families also felt strongly that they needed to engage, to ensure that they had ‘done right’ by their loved one. Families who received the service valued their MNISA attending reviews and investigations with them. MNISAs were able to explain, in simple terms, why the investigations were happening, what the process would involve, investigation timelines and who within the hospital was responsible for answering specific questions:

“One of the biggest stressors has been not understanding the information and not knowing who to ask or who to turn to with any questions. This is all an absolute minefield for anyone to understand that's not involved in healthcare. We don't know what we don't know—what's meant to be happening from start to finish and all of the middle. [Our MNISA's] been able to be that person to explain information in a way that we understand it, to know the correct questions to ask of the right people so that we're getting the answers that we want”.

(Mother whose baby died in the neonatal period, INT22)

MNISAs also attended meetings with families to ensure that they were taken seriously by the hospital and document what was said. MNISAs often helped hold the hospital accountable for what they told families, as this might change from meeting to meeting. One family described:

“Because [our MNISA] was at the original debrief and the next meeting, she heard they gave us two completely different accounts. The first saying that this was done wrong and this was done wrong. Then we go for the second meeting and them saying, “Oh no, actually you was misinformed.” We're like, well, we definitely wasn't because [our MNISA] was there. [Our MNISA] said to them ‘You have to understand this is completely different information from what the family's been given.’ I think even she was quite surprised”.

(Family whose baby had a brain injury and died in the neonatal period, INT18)

Furthermore, MNISAs supported families with practical and administrative aspects of these processes (e.g., reading reports, responding to emails, chasing updates/information and gaining access to medical records) when they, overcome with grief, found it difficult to process this information or to continue to fight the hospital for access to their information. Where required, they signposted families onto additional support services. Families reported that MNISAs provided an element of structure and understanding to an experience that felt disordered:

“She's been absolutely brilliant in that she made notes of everything. At this point my brain was ‐ to say it was working tangentially would be an understatement. I was having to re‐map my brain because nothing was working properly. She would take notes, get all the questions, get it all down. She's been amazing. I'd have had to spend 10 hours researching different acronyms and gobbledy‐gook they like writing on websites. It was very, very helpful.”.

(Father whose partner died giving birth, INT8)

There was only one instance where a family felt that their MNISA should have followed up with them more consistently during these processes. This was influenced by them feeling unsure about the next steps for their case and how/when their MNISA could be involved:

“[Our MNISA] explained her role and asked if me and my husband had any questions. But right at the beginning we didn't because we just didn't know. When someone's asking you, ‘What can I do to help you?’ it's like, I don't know how it's meant to go. Is it normal that the governance team to have disappeared? Is that normal? Or is it wrong? She did forget a few things, like type ups or copying [their husband] into emails. But I think we don't know how much she can do”.

(Mother whose baby was stillborn, INT5)

#### 4.2.2 Comprehension of Care

MNISA support helped families to understand their care and what happened to them (including lay explanations of technical language and review terminology). A key part of this was creating timelines of care: *“She wrote it down, captured everything and then emailed that to us—that was really useful. She took notes whilst we darted off in random directions. She put it in order for us, made note of our concerns and questions”* (Family whose baby had a brain injury, INT25). Where English was not a family's first language, this was especially important, as MNISAs could communicate their questions in English and facilitate accessibility:

“Because of my English language barrier, I wasn't comfortable enough to ask questions if the conversation was in English. So I told [my MNISA] every question I had, and she gave me the responses in writing. It would have been very different [without my MNISA] because I didn't even go to the hospital to ask for my medical report about what happened. It was very good for me to know where the mistakes were and what happened. Because I would have wondered all my life. I would have to regret what happened and ask questions about why.”.

(Mother who had an unplanned hysterectomy, INT15)

Families without MNISA support reported having no clear avenue to ask questions about their care and did not understand (1) what happened throughout their care, (2) what the next investigative steps would involve and, crucially, how to access the support that they needed. These families reported feeling alone at an emotionally overwhelming time and researching answers for themselves—notable in a description of feeling like *‘Dr Google’*:

“I was on my own researching pain management thinking, I know I'm on Oramorph here, is this normal? NHS Choices has nothing. I tried that, then started looking up the Royal College of Midwives—what do they offer? I looked at the NICE guidelines; I then started looking up in journals. I didn't want to use Dr. Google, although I was. It's not about MNISAs being an expert in sepsis, but actually knowing, this is the information we have on it; these are the experts. This is who we can push towards. This might help you get answers and if we don't get the answers, we'll look at other options or we'll feed it back. It's what I hoped would be there.”.

(Mother who was admitted to critical/intensive care and did not access the service, INT32)

#### 4.2.3 A Continuous Point of Contact

During an emotional time navigating complex investigation processes, where families often felt unsure of who to go to and how to raise concerns, their MNISA was their continuous point of contact—described as the ‘*golden thread’* (Mother whose baby was stillborn, INT19). Their involvement extended beyond a single dimension of care or an isolated process; rather, they supported with many different elements (e.g., answering questions, signposting and escalating concerns), which provided a sense of reassurance and continuity for families:

“With [our bereavement midwife] hers is more emotional help regarding the loss. When [our MNISA] came along, she's like a Jack of all trades. She can take so many things under her wing. [Our MNISA] actually actioned as well. I think we felt – not lost but nowhere to really turn when we've got questions about certain things. We'll ask people, that's not us, that's not ours, we can't answer that right now, or it's not my role to answer this. [Our MNISA] was that person, that could answer the majority of things that we've asked”.

(Father whose baby died during the neonatal period, INT22)

Family experiences (those who did and did not receive support) indicate that the value of advocacy was centred around providing ongoing support during a time of significant uncertainty—where there was a lack of clarity about who families could go to, how to access the support that they needed, ensure their concerns were escalated and their questions were answered.

### 4.3 Factors Influencing Ongoing Engagement With the Service

#### 4.3.1 MNISA Experience, Knowledge and Approach

MNISAs had varying backgrounds (clinical vs not clinical); however, families spoke positively about the experience that their advocate brought regardless of their background and any specific expertise. The key benefit associated with a clinical background was that MNISAs could provide a level of understanding that felt validating: ‘*She told me she [had a clinical background] and I instantly thought, ‘you get it’, you understand what actually happened’* (Mother whose baby who died in the neonatal period, INT1). Most importantly for families was the MNISAs' knowledge of the NHS, how the health care system worked (including processes that followed adverse maternal and neonatal outcomes) and their empathetic, family‐led approach. Families valued how MNISAs applied their knowledge, experience and seniority to support the individual needs of each family in a way that felt personal rather than procedural. These factors were viewed as essential facilitators by all families:

“Being compassionate is very important because you're constantly facing this medical and impersonal language and systems. That human touch is missing and it's quite brutal”.

(Mother whose baby died in the neonatal period and did not access the service, INT34)

“Her seniority helped us feel confident that the support we were receiving was thorough and well‐informed. She had a deep understanding of the system and the needs of bereaved families, which made her well‐equipped to advocate on our behalf and ensure our concerns were addressed. We knew she had the authority and experience to manage complex situations; provide the right resources and push for the support we needed”.

(Father whose baby died in the neonatal period, INT12)

### 4.4 Independence From the Hospital

MNISAs being independent from the hospital where families received their care, but still within the broader health care system, was an essential facilitator to engagement for all families: *“The fact that she's independent makes all the difference. I don't trust the hospital, I don't trust the director of operations, I don't trust maternity governors. I don't trust the consultant”* (Mother who was admitted to intensive care and whose baby was stillborn, INT31). Families felt that MNISAs maintained their neutrality and autonomy, effectively balancing a relationship with families and the hospital. Their independence prevented families from feeling that hospitals were marking their own homework and protecting their reputation:

“If it was someone within the trust, not an independent person, I think the feeling of them closing ranks, like I've had with the consultant, that fear would be very real. The fact I know [our MNISA] doesn't work or hasn't worked in this area, she's not going to be friends with the person that didn't prescribe me the antibiotics.”.

(Family whose baby had a brain injury and died in the neonatal period, INT13)

Such independence was central in differentiating MNISAs from existing maternity roles (e.g., bereavement midwives). Unlike existing roles, families felt that MNISAs were separate from the hospital and provided them with an avenue to speak freely and honestly about their experiences, without fear of implications for their future care or investigations:

“Although bereavement midwives are there, they're still very much associated with the hospital. They're still protecting the reputation of that hospital, whereas with the advocate I was able to have open, honest, transparent conversations with her. I've been able to sound out ‘Actually am I going crazy or is there stuff that's missing?’.”.

(Mother who was admitted to intensive care and whose baby was stillborn, INT30)

## 5 Perceived Impact of MNISA Support

### 5.1 Psychological Impact for Families

#### 5.1.1 Alleviating the Emotional Overwhelm That Families Experienced

Families described how MNISA support provided space for them to grieve. MNISAs alleviated the pressure, guilt and uncertainty that they experienced when going through investigations to obtain meaningful answers—to feel that they had done right by their loved one. MNISAs supported families to rationalise and approach tasks that felt too overwhelming to approach alone. For one family, MNISA support helped them to engage with the investigation, as their advocate made the process more manageable. Without this support, they described balancing the importance of preventing future harm with the emotional impact of the investigation prolonging their grief:

“I was able to just grieve and go through the process of grief not worrying. Because you feel like you're letting your baby down if you don't advocate for them yourself and you're like, I can't just let her death be in vain or let it just happen. I feel like a bad mum if I do that and so [our MNISA] taking that burden was amazing.”.

(Mother whose baby was stillborn, INT19)

“It was that bad before [our MNISA] came along. I kept saying to [my partner] ‘I don't want to do the investigation’ because it's so overwhelming that I couldn't bear it. We weren't understanding anything, we had questions, but the questions couldn't be answered. It felt like it was just prolonging the grief rather than actually helping, which is the point of the investigation ‐ to make sure that things don't happen in the future. It was affecting us in such a negative way, it just felt like so much work when we didn't have the energy to breathe. She has just done all of that for us.”.

(Mother whose baby died in the neonatal period, INT22)

Families also felt overwhelmed as the world carried on around them—managing grief and investigations while returning to work, being present for their children and prioritising their relationships. The support provided by their advocate allowed families the ability to get back on their feet. For fathers/wider family members, MNISAs were particularly important in providing them with reassurance that their partner/loved one was supported, if or when they themselves felt they could not. Many families therefore emphasised that by alleviating the overwhelm that they experienced, MNISAs had a positive impact on their psychological well‐being:

“I'm on the sidelines trying to support [my partner]. But a lot of the times I can't be there, but [our MNISA] is there. I'm so thankful to her to just have someone to assist me in looking after [my partner]. It would have been really tricky for me to go back to work as I always did. Then where does that leave us?”.

(Father whose baby died in the neonatal period, INT27)

“Having somebody that's able to advocate and explain was absolutely valuable. I'm not going to say that I would have had a nervous breakdown without her. But the chances of me having a nervous breakdown would have been significantly higher without my MNISAs input, help and support.”.

(Father whose partner died giving birth, INT8)

### 5.2 Enabling Family Voices to Be Heard

#### 5.2.1 Feeling Listened to and Validated

MNISA support enabled families to feel actively listened to. Many families who previously reported feeling ignored described their engagement with their MNISA as the first time they felt listened to, but also validated and believed:

“The advocate changed our entire outlook on the process. It was the first point that we actually felt heard because we didn't ask for this. We didn't ask for any of this process to be triggered”.

(Father whose baby had a brain injury, INT28)

“Although we had our voices, it felt very much like we were silenced. [Our MNISA] provided us almost like the pedestal to be heard and that's all we wanted, to be heard, to be listened to, just to be validated and believed”.

(Mother whose baby died in the neonatal period, INT24)

#### 5.2.2 Ensuring That Family Concerns Are Acted Upon

Families' grief and emotional engagement with the incident meant that they sometimes struggled to take a step back so they could formulate their concerns. To enable family voices to be heard, MNISAs not only actively listened to families but also supported with framing their questions and concerns into actionable points, which could be fed back to the hospital and included in investigative processes. MNISAs were able to provide empathetic but objective support, to collate and effectively relay family experiences, questions and feedback:

“She said, ‘Send me every question you possibly have, I'll put a letter together.’ She used her notes she'd taken from our story and all my questions. When I read it, I was like ‘Oh my God, this is what I've been thinking but I can't say’. I would have never been able to put it into words like she did, I just cried. Because she's written down exactly how I'm feeling and [the hospital] needs to know about it”.

(Mother whose baby had a brain injury, INT2)

Furthermore, families reported that MNISAs knew who within the hospital was the right person to voice their concerns to, which could enable them to be heard. One mother, who had the unique experience of navigating investigative processes with and without an MNISA, recounted that their MNISA's involvement enabled her to feel listened to where it mattered (i.e., Trust‐wide):

“Our voice would not have been heard without [our MNISAs] involvement. The connections she had are how we ended up meeting with the right people to then have the conversations. Without that role, I felt very much like I'd tried everything. I couldn't…I just was not heard in any way. In the bigger…you know? Trust‐wide, in the place where it mattered, I wasn't heard.”

(Mother who was admitted to the intensive care unit, INT6)

By listening to, believing and validating families, MNISAs served as the mechanism that enabled their voices to be escalated and heard.

### 5.3 Examples of Family‐Led Changes

There were nuances in family perspectives of change, as voicing a need for system‐level change was not seen as applicable or relevant for every family. For some families, this was not their current focus (though could be in the future); for others, they were too recently bereaved to know what change to advocate for, or they felt that the context of what happened to them was not avoidable:

“My view is that we won't be one of those families that drive change or be affected by [reviews] because our issues fall outside of that jurisdiction. I don't think our story necessarily drives change within the hospital. Sometimes, unfortunately, terrible things happen outside of everyone's control.”.

(Mother whose baby was stillborn, INT20)

Families also acknowledged the complexity of influencing change in health care and maternity and neonatal care specifically, where it takes time and there can often be a reluctance to change—creating barriers to overcome before family feedback can escalate to evidenced change:

“[MNISAs are] up against a wall. What they're trying to fight is unviable. I see and hear that they're trying their best, plugging away, trying, but the system is so broken, it's an unfightable battle”.

(Mother who was admitted to intensive care and whose baby was stillborn, INT30)

However, families provided examples of organisational changes and routes for change, already achieved or planned. This involved MNISAs listening to family experiences, which changes were needed and feeding this back to the hospital, thus serving as a mechanism to help facilitate change. Families emphasised that their feedback was led by them and MNISAs did not tell them what they should think, say, write or ask for. Changes primarily related to (1) upholding standards of care and/or making improvements to existing practice (e.g., putting protocols in place, reviewing equipment and providing compassionate bereavement care) but also (2) making new and innovative changes (e.g., co‐producing leaflet for risks of pre‐term birth and introducing a new voluntary Trust service (for in utero transfers) (Table 4)). All were driven by the aim of preventing avoidable harm and not wanting what happened to them to happen to another family.

**Table 4:** Examples from families illustrating changes.

Type of changes		Case study examples & illustrative quotes
*Upholding standards of care and/or making improvements to existing practice:* In many cases, families did not describe making new changes to the system following the serious harm or death of a mother or baby. Instead, they reported how the harm that they experienced could have been prevented if existing guidance or standards of practice were followed and/or small improvements were made.	Staffing and resources	A mother who experienced an unplanned hysterectomy described that as a result of what happened to her and feeding back to the hospital: *“There's going to be one more nurse in the room so they're physically able to check how much blood is lost before proceeding with any other procedures. [With me] they didn't actually check that. Specific action recommended to the labour ward team was to have two maternity support workers available in the obstetric theatre, concentrated on assisting the scrub nurse to calculate the main blood loss”* (Mother who had an unplanned hysterectomy, INT15).
	Procedural changes to ensure that care guidelines and/or review processes are followed	One family described how their MNISA helped them escalate concerns about guidelines not being followed during their care and advocated for future compliance: *“She's already gone to the Trust board and things. But not even just the Trust board, but the regional Trust board and things like that and spoken about us and [our baby]. They said they were going to implement the fact they should use an ultrasound machine during labour when they don't know where the baby's head is, even though that was in the guidelines since like 2020. But it's only just now they're saying that they're going to do that. But it's too late for us”* (Family whose baby had a brain injury and subsequently died in the neonatal period, INT18).
		Another family whose baby was stillborn described how the MNISA help put in place a protocol when parents want to take their baby from the hospital: *“We had to fight quite a lot to be able to not leave our baby at the hospital. And for him to be able to go straight to the funeral director. Which is now a change that they put in place. They've now actually got like a protocol for that. So that's now an option”* (Mother whose baby was stillborn, INT21).
		One family described how their MNISA supported them to obtain an accurate and correct PMRT report, highlighting where their report needed to be re‐reviewed: “*They asked us do you want a PMRT and we said yes. They asked us do you want an HSIB report and we said yes because we were not expecting anything bad to come out of it. We were only expecting that there would be minor issues and we might make it better for future children. The Trust reiterated everything…. [they] stand by the same things said that night. The CTG's were normal and there was no complication. Nothing would have changed the outcome. In the HSIB reports it said that the CTG's were abnormal. And when we asked the PMRT to be re‐reviewed the outcome was a D. I think is the worst outcome and we never received the report from the PMRT. Then [our MNISA] requested that the PMRT should be done again because she thought the PMRT was not done properly. So it was [our MNISA] who got us a proper PMRT. We were given a redacted copy of that, but again they had a meeting with us in which they read the whole report, but the copy that they gave us was a redacted copy. Again, it was such complex document that we could not understand much of it. But we know the essence. So there were two PMRT's. One was the outcome B and one was outcome D”* (Family whose baby died in the neonatal period, INT29).
	Compassionate bereavement care	One family described how they had multiple negative experiences of their bereavement care and wanted this to be raised to ensure that future practice could be improved, which their MNISA supported with. They provided numerous examples (including interrupting/rushing them while spending the last few moments with their baby, getting their baby's name wrong, having to leave the hospital past the labour wards and not having adequate bereavement care resources):
		*“Just saying we weren't happy, it kind of made us feel like we'd done something wrong. We – just spoke up to make sure nobody else had to go through what happened to us. So, when we were getting ready to leave, we were going to carry him out and give him to the funeral director in the car, but she kept sort of coming into the room, as though she was sort of rushing us. Our family were sort of outside the door, and she was getting quite agitated with them that we weren't being a bit quicker. We just wanted that final bit of time. Then rather than giving me the injections that I needed to go home with, rather than just giving them to somebody outside or just waiting, she came into the room and disturbed us again and then told us to make sure that we covered up his [their baby's] face before we left because we didn't need to be upsetting anybody”*.
		*“We weren't allowed to leave from the front and actually, it's one of those that it probably would have been hard to leave out the main doors but also, the option should have been there if that's what we wanted to do at the time. Unfortunately, there were two other couples going through the same as what we were, the same weekend. So the bereavement rooms they had were then obviously busy, which is not their fault at all, but it meant we were on the labour wards, so we were hearing babies crying, which is okay but it's just one of those that you have to deal with. We were on the main labour ward, and you'd think oh okay, can we leave through the main doors, but no, we had to go through a back way and out the back door”*
		*“[Our bereavement midwife] kept coming in and kept on getting his name wrong as well. She kept on calling him [a different name]”*
		*“The time that we should have had with him should have been special, but it just had effects on us that were upsetting. We did the hand and footprint, but they didn't quite have the right clay did they? Or no one really knew quite how to use it properly and heat it up properly. So we haven't obviously got the best prints have we? Because we were told when we went in to be induced that actually, they had quite a few stillbirths around the same time. So they were trying [to make what was in a memory box], but they didn't actually like have a box ready. That's something they're going to change now to make sure they've got enough stock and actually make sure that there would be a box available”*
		*[Our MNISA] helped get all that in place. Then from the PMRT, it's been put into place about checking on stock, training for staff. And [our MNISA] is now carrying on and she's noticed common themes coming up and she is now taking that on board. She's now pushing to make it that birthing times are now done earlier on in your pregnancy. She's made it so that it wasn't just for us personally getting the PMRT report and getting changes in place. It's now carrying on to lots of people who want to see these changes now. It's amazing to think in a way we're a part of that”*
		(All quotes from the same interview ‐ Family whose baby was stillborn, INT21)
	Family‐facing information	A father whose partner died giving birth described how working with their MNISA helped to remove an outdated sign stating that partners could not sit on the beds. This prevented them from being able to comfort his partner when waiting for a C‐section: “One of the things that [our MNISA] has done, in the waiting room before we went in for the c‐section, there was a sign that said the beds are for the use of the patients only. I took that to mean that I couldn't get in bed and cuddle [my partner] which I wanted to do. She was stressed out of her mind, and we had to wait for 3 or 4 hours while they were waiting for the on‐call team to come in and all this sort of sh*te. I could have been there cuddling and that would have been the last time I got to cuddle her, and I couldn't because I was playing by the rules. I mentioned that to [our MNISA] so she's helped get that sign removed. It's one of them, it's a small idiosyncrasy in a way but that, if I'd got to have that last cuddle, be able to calm her down when she knows she's about to have a big surgery” (Father whose partner died giving birth, INT8).
		One family described how their MNISA supported them to escalate concerns regarding the lack of clear and accessible guidance for using the cold cot – to ensure that families can comfort their baby and use the cot correctly: *“We weren't told how to use the cold cot properly and that's something we've now read about. We said even if they just put like a leaflet or like a laminate in the cold cot to say how to use it that would be better than nothing. Because the minute they [staff] walk out of the room, you forget everything they've just said to you. And they didn't say to us how often we needed to put [our baby] in, for how long. I wrapped four blankets around him and then put him in the cold cot, because I didn't want him to be cold. It was only when we had another midwife come in that was just, she was amazing the whole time we were there, she said “when you put him in, just make sure you put him directly on it and then if you want to put a blanket on, you can put it on top.” Like th knew why I was putting a blanket on him, and she just told me how to do it in a different way that was obviously still going to benefit him, but I could still be his Mum and cover him up with a blanket. She just understood exactly. So, we said [to our MNISA] that needs to be more clear”* (Mother whose baby died in the neonatal period, INT1).
	Appropriate care facilities	One family described how, based on their feedback, their MNISA is now collating feedback from families more widely on their experiences of the bereavement suite and the appropriateness of where it is located. Learning from example cases where bereavement suites demonstrate good practice, to inform local improvements: *“The bereavement room was outside of the [delivery suite] so they said well that's not appropriate because we want the security of feeling like no one can just go straight in. So they said look, this room's not appropriate and it's not fit for purpose, we need to move this. So a big thing [our MNISA] is doing is speaking with families and asking whether they would give any feedback if they were in the bereavement suite themselves. [Our MNISA] is speaking with a lady who works at this one particular hospital, that has awards for the best bereavement suite in the country. [Our MNISA] is doing a really good piece of work with this lady and working to find out what's best to go in the bereavement suite at our hospital. To make sure the room is right. So yeah, she's moving mountains in a short space of time, honestly, I cannot explain to you how incredible this lady is”* (Mother whose baby died in the neonatal period, INT4).
		One family described the equipment changes that the MNISA was able to push for and ensure that the hospital put in place: *“[The hospital] have done a full equipment upgrade, or at least they say they're going to, on stuff like SATs probes and resuscitators. And they now say they're going to do more checks on emergency drug trollies that I don't think they had before”* (Father whose baby died in the neonatal period, INT27).
		Another family described how their MNISA made sure that for their second pregnancy, they did not receive care from the same clinician where there was a breakdown of trust: *“What happened was that when we had the booking with like the initial scans with our next baby, they gave us an appointment with the same obstetrician that came and met us on the same night. We think she was not very honest with us, and they gave us that same doctor' when we have publicly, openly said that we don't want to see her again. They made the appointment with her. Maybe it was a clinical error or a system generated appointment, but it was a big, big error and we didn't know how to sort it out. So we just texted [our MNISA] and she took care of it. Within a week we got a new appointment with the doctor we've requested by name. So [our MNISA] did that for us, within less than a week it was sorted”* (Family whose baby died in the neonatal period, INT29).
	Facilitating access to additional support	One family described where their MNISA has highlighted an increased need for culturally appropriate psychiatric care, through advocating for their needs following the death of their baby: *“The support we received from the MNISA highlighted some important areas where improvements could be made, especially in terms of communication, cultural sensitivity and ensuring families feel heard and supported during difficult times. The level of care [our MNISA] provided by advocating for us, addressing our concerns and pushing for support like a Tamil‐speaking psychiatrist suggests that there may be room for increased focus on the emotional and cultural needs of families facing similar situations [in the future]”* (Family whose baby died in the neonatal period, INT11).
		One family described how their MNISA supported them in receiving ongoing mental health support and also identified a gap in mental health provision for severe cases, which was escalated further: “*Our MNISA rung round every single person, organisation, and charity to get me mental health support. And I asked, have you ever struggled so much to get support for someone? Is this a me thing? Or is this an everybody thing? It turns out it's a me thing. We noticed there was a gap in the NHS service and in the charity services. I was extremely high risk and because I was so high risk, they didn't want to take me on. So I struggled to get support because of that. But when I had a few bad episodes, [our MNISA] referred me through to the crisis team. She also tried to get help for me in the community. Unfortunately, again, another door just shut in our faces. And this is something we're taking to the ICB – to say they need to look into their mental health services and quite how they're allowed to put criteria in place that basically means that they will only deal with people they feel they can manage, but not with the one's they can't manage. To say they're not going to do anything for those people. It isn't right. Our MNISA referred us for the first and second time, but they didn't go through. So she'd done a third referral and that one went through. But then I had to do an interview to decide if they would take me on. [Our MNISA] said that if I didn't get in this time, we would be taking it to ICB level. So she's really done everything she can in that regard”* (Mother whose baby died in the neonatal period, INT4).
*Making new and innovative changes:* In some cases, families worked with their MNISA to facilitate new and/or innovative change. These changes were informed by their experiences and were described as changes that could have prevented the serious harm or death of a mother or baby, had they been implemented sooner.	Introduction of new voluntary NHS Trust service	Based on their experiences, one family was able to identify the gaps in the process for in utero transfers between hospitals. With the help of their MNISA, this family was able to escalate their concerns to the right person in the hospital to improve the service in the future: *“We'd spoken to [the hospital] and we said why did this happen? Why was staffing so low? How can we improve this? What are you doing as a hospital to work towards making sure this doesn't happen again? That's when I said look, surely there could me a volunteer service where retired midwives or midwives that have gone into a different path or career and people that have the ability to do the transfer and want to volunteer to do the transfer if needed. Because we do know that there's a plan A, which is the midwives on the board, plan B which is the community midwives that would then get called out to afterwards. But I said, well, where's plan C? When all of those people are busy, where's plan C? [Our MNISA] managed to get us into the right meetings with the right people to discuss that service and [the hospital] agreed that it was needed even if it may be a rare situation. So if that rare situation comes about, there's now something in place to make sure that no emergency transfer is ever refused because now it's fully staffed. This service has been named in honour of our son. And now our MNISA has got the right environment to spread this idea further. Like she's just done one the other day at some kind of medical meeting, where she was able to anonymously explain about [our son's] journey and the volunteer service that's been set up. Which as you can imagine, is now making it widely spread so others can pick up on it”* (Mother whose baby died in the neonatal period, INT4).
	Introduction of new pathway changes	A bereaved mother described how her experience of pre‐term birth led to the co‐development of a preterm birth leaflet, which detailed the symptoms that pregnant women should look out for. These were symptoms that she described could have saved her baby's life: “*I said [to our MNISA], “why's there no preterm birth workshop?” You know, somebody should have sat down and said, “You're at risk of preterm. I can't tell you how preterm, but we do a morning session of any signs or symptoms”. Our MNISA took that back to the Trust and one of the outcomes was that they developed a preterm birth leaflet. We were given it with the final copy of the investigation report, and given it to review it, like “do you think this is good enough? Does this explain things?” and we were just absolutely distraught because when we looked at it, there were six symptoms of preterm labour and out of the six, I expressed four of them in the weeks and hours leading up to [our son's] death. So I was just like, “Oh my god. This could have saved [my baby's life]”* (Mother whose baby died in the neonatal period, INT24).
		A bereaved father described how they worked with their MNISA to escalate adding a flag to patient systems to indicate when they are on a pre‐term birth pathway: *“They're meant to have put a flag on patient's system, on patient's notes to say they're on a pre‐term pathway or they're on consultant‐led care for X, Y, Z. So basically when ladies like [my partner] that are on these, sort of, consultant‐led pathways, the idea is that they phone triage, and they get seen straight away. But then when we phoned triage, the nurse didn't access the systems for hours after anyway, so you could say that's a good fix”* (Father whose baby died in the neonatal period, INT27).
		A bereaved mother provided examples of different changes related to their experiences of care, which their MNISA had supported them to escalate to the hospital and follow‐up:
		*“One of the outcomes [from report outcomes MNISA supported to chase and action] was this unconscious bias training needs to be delivered. They did their first ever maternity unconscious bias training last year, off the back of what we went through in 2024. Which, it worries me – should that not be engrained in training anyway? I did an interview like this where they recorded me and played that on a screen to 75 staff members. Then I worked with the chief midwife and chief obstetrician in helping them to sort out a racism and unconscious bias module across maternity services”*
		*“One of them was, this is going to sound ridiculous but putting up labels on nurses stations to check patients records before they attend, because they didn't check my records before I attended that week, to see multiple presentations.”*
		*“They've also established a new telephone number for paramedics to be triaged to the right place, because one of the things they've said is I should have been taken to maternity assessment, I should have been taken straight to delivery suite. But again, my thinking with that is I wasn't in labour. It's something about having a senior person and make sure they're listening fully to what the paramedics are saying. So actually if someone's crashing like I was, when my lips are turning blue, there's a full picture there and we send them to the right place. So that telephone number has been implemented but the notes on nurses stations still hasn't [in progress]. Subsequently, through the Facebook groups and everything, I've found out that someone had multiple presentations six months before us, baby passed away and they were supposed to do a lot of the changes then and they hadn't. Had they, we would haven't have been in our position. There's so many missed opportunities and for me, there is something about, they could say it, but I want to see it done. [Our MNISA] helps me to say to them, well is it done? She pushes, every time we debrief she's like I've asked them about all of these things. Because when your look at it like that, you kind of go how many other babies could have been saved had you acted in accordance with your own PMRT findings and actions?”*
		(All quotes are from the same interview – Mother who was admitted to intensive care and whose baby was stillborn, INT30)

### 5.4 Future of the Service

All families felt strongly that the service should continue and felt concerned for others in similar situations to their own if the service was discontinued:

“It's the only bit of funding within the health service in this country over the last god knows how many years that you can actually say ‘Christ, they've funded something that is making a positive change. If anything they need more resources and funding’”.

(Father whose baby died in the neonatal period, INT4)

“It's the question of are MNISAs worthwhile? If it's somebody's call to make and they decide they're not, then shame on them because people that are going to become me and [their partner] in the days, years to come; if they don't have an advocate and they've been treated as we feel we've been treated, then there is serious trouble, very serious, mental, physical, I honestly don't know how they'd do it”.

(Father whose baby died in the neonatal period, INT27)

## 6 Discussion

### 6.1 Key Findings

Family experiences suggest that MNISAs met proposed service aims. All families expressed an unequivocal view that the service should continue. Families felt that MNISAs listened, validated their experiences, provided support with navigating investigative processes and helped them to comprehend their care. In a fragmented system, the support provided was continuous, which helped to alleviate the emotional overwhelm that families experienced. Lack of knowledge about MNISAs, timing of the offer and distrust in the hospital created initial hesitancy towards accessing the service, alleviated by a clear explanation from their MNISA. Families valued MNISAs' empathetic approach and knowledge of the health care system, with independence from the hospital being an essential facilitator to ongoing engagement. By listening to their experiences, MNISAs enabled family voices to be heard by the hospital. This created a foundation for MNISAs to help facilitate family‐led, organisational‐level change, driven by the importance of preventing future avoidable harm. Families who did not receive the MNISA service indicated that they would have wanted and valued independent advocacy support.

### 6.2 How Findings Relate to the Existing Literature

The distrust that families described when being offered this support mirrors existing evidence which demonstrates that experiencing adverse outcomes in maternity [5, 8, 9] and health care more broadly [39] can result in distrust in health care services [5, 8, 9]. Although MNISAs understood their role and could explain it, communication from wider stakeholders led to uncertainty about the service. The service is clearly valued; therefore, clear and consistent messaging about how MNISAs can support families is essential to build trust and prevent accessibility barriers. This aligns with research showing that conflicting information can reduce trust and trigger reluctance to engage with health care services [40]. It is also important to consider the scale and impact of grief, acknowledging that families may require accessible ongoing or open offers of advocacy support [41].

The findings support research showing the complexity and volume of reviews, with often limited communication about what is involved, expected timelines, how families will be supported and how they should escalate concerns/future learning [5, 42]. Aligning with patient‐safety literature [43], the findings demonstrated that families were unsure about investigative next steps and who to turn to. Some families reported that they would not have engaged with investigations without their advocate. This highlights an ongoing gap in family support to engage with these processes, despite evidence showing that family engagement is essential [5, 22]. Wider evidence notes the importance of streamlining and simplifying patient engagement [44] in care and investigations. In a maternity setting, a point of contact who can answer questions, liaise with services and provide updates has been deemed essential [5, 22] and MNISAs fulfilled this role for families.

MNISA support aligns with similar roles that employ advocacy principles (e.g., active listening [28, 29, 45] and supporting in an empathetic, person‐centred way [28, 29, 30], understanding concerns and advocating [29, 30]). These attributes are central to delivering an effective advocacy role [29, 30]. The findings also suggest that the two service aims were complementary; MNISAs needed to listen to families and understand their experiences, to support them effectively and facilitate family‐led change. Families reported that change was not easy or quick, with notable barriers, including a systemic reluctance to be open‐minded and learn from adverse events [46]. Families may not be aware of further, system‐level (i.e., national) changes; yet, the findings show that many families felt heard by the hospital because MNISAs escalated their concerns.

Some characteristics made the MNISA service unique for families, compared to other roles (e.g., bereavement midwives), for example, independence, which enabled trust to be established and allowed families to feel that they could speak freely. This supports research on the wider perceived benefits of independent advocacy (e.g., reducing bias and amplifying service‐user voice in mental health settings) [30, 47], strengthening the rationale for the importance of independence in contexts where a breakdown in trust is notable. Furthermore, MNISAs were able to provide continuous support, suggesting that they enabled a sense of continuity that families had not previously experienced. These benefits support research on continuity of care in midwifery, where these models can help families feel listened to, safe and empowered to contribute to their care [48].

### 6.3 Policy and Practice Implications

The findings [33] were shared with the national maternity and neonatal review team [10] to inform future learning. Family experiences of feeling silenced are notable, but not new findings [8, 9], and the importance of listening to families remains a central part of review recommendations [8, 9] and government initiatives [10, 11]. The findings demonstrate that an independent advocacy role could be a mechanism for achieving this, as focus should be placed not only on the importance of listening to families but also how their concerns are acted upon in practice. MNISAs are uniquely placed to provide continual support, filling a gap during investigative processes [5]. Independent advocacy services could be implemented across other health settings where patients may experience harm and a subsequent breakdown of trust [39]. However, policymakers would need to consider the balance with cost implications and the need for ongoing evaluation.

Recently published interim reflections from the national review [11] highlighted 19 issues consistently reported in maternity care. Notably, findings show the potential for MNISAs to support with some of these issues (e.g., lack of empathy resulting in women feeling blamed or guilty; lack of family engagement in reviews; failure to protect families from hospitals ‘marking their own homework’; challenges accessing medical notes—or these being redacted later; families being placed in inappropriate spaces, e.g., on wards with newborns after experiencing a loss; having to liaise with multiple contacts; lack of support for the long‐term impact of adverse outcomes; and overly legalistic and complex approaches) [11].

### 6.4 Strengths and Limitations

This study is the first to evaluate family experiences of an independent maternity and neonatal advocacy service across England. Our approach aligned with previous rapid research [35], and we took additional steps to maintain academic rigour (e.g., peer review of study protocol by experienced qualitative researchers, oversight from a study advisory board throughout the evaluation and PPIE input as part of the project team) [31]. However, the sample may be biased towards families who had positive experiences (due to the recruitment approach), and we were only able to recruit three families who did not access the service. To help minimise bias, study information was shared with all families that MNISAs were supporting, interviews and written responses asked open‐ended questions to explore experiences and recruitment of families who did not access the service used varied approaches (e.g., via Sands, maternity and PPIE groups) [31]. Although we interviewed some families from minority ethnic backgrounds and families whose first language was not English, the sample was predominantly White British. This lack of diversity mirrors the wider accessibility patterns observed in the MNISA pilot and does not reflect the broader population of families affected by adverse outcomes [33]. We did not use a theoretical framework, as an inductive approach enabled the nuance and breadth of family experiences to be captured.

### 6.5 Future Research

Further research is needed to understand how independent advocacy can be embedded into maternity and neonatal care, particularly the longer‐term impact [33]. Future research should explore barriers to access and potential inequalities in who receives advocacy support, implementation, staff experience and cost [31, 33]. Families provided examples indicative of system change, but further research is needed to understand how such changes contribute towards preventing future avoidable harm and widespread national learning.

## 7 Conclusions

This independent advocacy service was implemented during a complex and evolving period in maternity and neonatal care, amid national reviews. Family experiences suggest that MNISAs met service aims. An independent advocacy service in maternity care provides an avenue to listen to families but also enable them to be heard and their concerns meaningfully acted upon. Further research is essential to understand the sustained impact of independent advocacy on family experiences, how the positioning of these services can facilitate equitable access and how examples of change may contribute towards preventing avoidable harm.

## Author Contributions

**Rachel Lawrence:** conceptualisation, methodology, investigation, validation, data curation, writing – original draft, writing – review and editing, formal analysis. **Julie Hartley:** conceptualisation, methodology, investigation, validation, writing – original draft, writing – review and editing, formal analysis, data curation. **Nadia Crellin:** conceptualisation, methodology, investigation, validation, funding acquisition, writing – review and editing, data curation, supervision. **Nina Hemmings:** conceptualisation, methodology, investigation, validation, writing – review and editing, data curation. **Holly Walton:** conceptualisation, methodology, investigation, validation, funding acquisition, writing – review and editing, data curation, supervision. **Emma Dodsworth:** conceptualisation, methodology, investigation, writing – review and editing. **Sarah Fisher:** conceptualisation, methodology, investigation, writing – review and editing. **Naomi J. Fulop:** conceptualisation, methodology, investigation, funding acquisition, supervision, writing – review and editing. **Saheli Gandhi:** conceptualisation, investigation, project administration, writing – review and editing. **Kevin Herbert:** conceptualisation, methodology, investigation, writing – review and editing. **Sonia Macleod:** conceptualisation, methodology, investigation, writing – review and editing. **Cate Maddison:** conceptualisation, methodology, investigation, writing – review and editing. **Raj Mehta:** conceptualisation, methodology, investigation, funding acquisition, writing – review and editing. **Stephen Morris:** conceptualisation, funding acquisition, supervision, methodology, writing – review and editing. **Pei Li Ng:** conceptualisation, investigation, funding acquisition, writing – review and editing, project administration. **Chris Sherlaw‐Johnson:** conceptualisation, methodology, investigation, funding acquisition, writing – review and editing. **Ben Wills:** conceptualisation, investigation, methodology. **Jenny Shand:** conceptualisation, methodology, investigation, validation, funding acquisition, writing – review and editing, supervision.

## Conflicts of Interest

Professor Jenny Shand is Non‐Executive Director at Care City (June 2019‐present), Advisor to the Harley Street Health District (Sept 2024 – Present), Senior Associate at ZPB Associates (February 2024‐ present) and an RSA fellow (Dec 2019‐ present). Professor Naomi Fulop is currently a Non‐Executive Director at Covid‐19 Bereaved Families for Justice UK (from August 2022 to present), a Member of the National Emergencies Trust Advisory Board (September 2025 ‐ present), an NIHR Senior Investigator and is supported by NIHR Central London Patient Safety Research Collaboration. She has also been a Non‐Executive Director at Whittington Health NHS Trust (Oct 2018 to Oct 2024), Trustee with Health Service Research UK (to November 2022), and formerly a member of the following: the UKRI and NIHR College of Experts for Covid‐19 Research Funding (2020), the NIHR Health Services and Delivery Research (HS&DR) Programme Funding Committee (2013–2018) and the HS&DR Evidence Synthesis Sub Board (2016). Professor Stephen Morris is currently (2022‐) a member of the SBRI Healthcare panel. His post is funded in part by RAND Europe, a non‐profit research organisation. He is also Director of Applied Research Collaboration East of England (NIHR ARC EoE) at Cambridgeshire and Peterborough NHS Foundation Trust. Professor Morris was formerly a member of the NIHR HS&DR Programme Funding Committee (2014–2016), the NIHR HS&DR Evidence Synthesis Sub Board (2016), the NIHR Unmet Need Sub Board (2019), the NIHR HTA Clinical Evaluation and Trials Board (2007‐2009), the NIHR HTA Commissioning Board (2009‐2013), the NIHR PHR Research Funding Board (2011‐2017) and the NIHR PGfAR expert sub‐panel (2015–2019). He was also an associate member of the NIHR HS&DR Commissioned Board (2014–15) and an associate member of the NIHR HS&DR Board (2015–18). Cate Maddison is a member of the THIS Institute Maternity PPI Panel, RCOG Women's Network and Scientific Advisory Committees and the NW Neonatal ODN Parent Advisory Group. Raj Mehta is Trustee of the Middlesex Association for the Blind (April 2015‐present; Chair since December 2020); Trustee of the Research Institute for Disabled Consumers (October 2018‐present; Vice‐Chair since August 2020); Non‐Executive Director of Evenbreak (February 2016‐present); and Trustee of the Thomas Pocklington Trust (November 2019‐present).

## Supporting information

Supporting File

## Data Availability

The data that support the findings of this study are available on request from the corresponding author. The data are not publicly available due to privacy or ethical restrictions.

## Endnotes

1 INT1 = Interview number 1
