# Supplementary material for: “That's All We Wanted, to Be Heard, Listened to, Just to Be Validated and Believed”: Family Experiences of Advocacy Support in Maternity and Neonatal Services
Source: Health Expect. 2026 Jul 3;29(4):e70750. doi: 10.1111/hex.70750 (PMC13332122; doi:10.1111/hex.70750)
Supplement: Supplementary file 1 — Supporting File [file HEX-29-e70750-s001.docx]

# Appendices

## Appendix 1. List of Abbreviations

- MNISA: Maternity and Neonatal Independent Senior Advocate
- ICB: Integrated Care Board
- PPIE: Patient, Public Involvement and Engagement
- NHS: National Health Service
- PSIRF: Patient Safety Incident Response Framework
- PMRT: Perinatal Mortality Review Tool
- MNSI: Maternity and Newborn Safety Investigations
- HIE: Hypoxic-Ischaemic Encephalopathy
- NIHR: National Institute for Health and Care Research
- RSET: Rapid Service Evaluation Team
- DSH: Data Safe Haven
- RAP: Rapid Assessment Procedure Sheets
- PSIIs: Patient Safety Incident Investigation
- NICE: National Institute for Health and Care Excellence
- CTG: Cardiotocography
- HSIB: Health Services Safety Investigations Body
- C-section: Caesarean section

## Appendix 2. Summary of pilot implementation


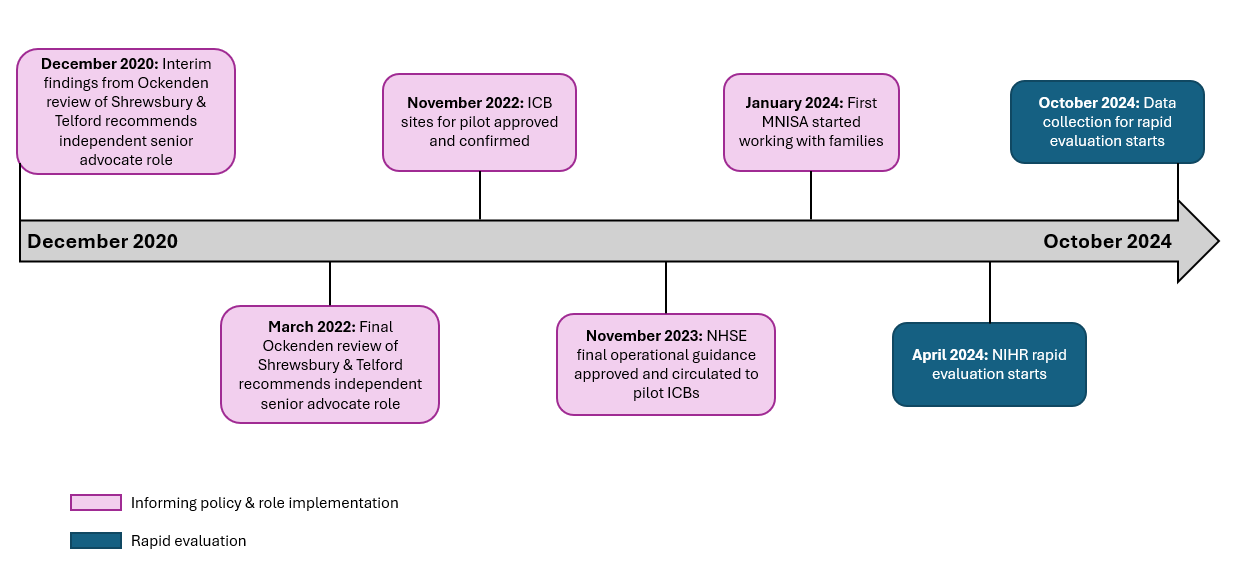


## Appendix 3. MNISA background and experience

| **Model of employment** | |
| --- | --- |
| Employed by ICB | 13 |
| Employed by external organisation | 2 |
| **Type of experience** | |
| Clinical^a^ | 4 |
| Non-clinical^b^ | 8 |
| Mixture of both clinical and non-clinical | 7 |
| *Note:*  a) Examples of clinical background include: Midwife, neonatal paramedic, nurse  b) Examples of non-clinical background: Advocacy, counselling, patient safety/governance, NHS systems change | |

## Appendix 4. Safeguarding protocols

| **For families** | **For staff** | **For researchers** |
| --- | --- | --- |
| Flexible participation (mode, number of interviewees, time and receiving interview questions in advance) | If staff express a wish to whistle blow or speak up – asked to refer to their local policy, signposted to National guardian’s office and encouraged to contact local guardian | Regular check-ins to debrief and ensure they are supported |
| Prompt support availability for day of the interview | If participants became distressed during interview, signpost to Sands helpline which also support staff | Signpost to relevant support services as appropriate |
| Pre-interview chat (not recorded and facilitated by Sands) |  | Attendance at publicly available training provided by Sands |
| ***Interview (facilitated by Sands):***   - Breaks throughout - Follow distress protocol where appropriate - Escalation if any safeguarding issues arise - signpost to complaints procedures at trust/ ICB where appropriate or needed |  |  |
| ***After the interview:***   - Check if participants are happy for researchers to follow-up/check in - Provide debrief form and verbally debrief - Check families have support in place - Refer to Sands helpline directly or other charities (e.g., Peeps, Petals), GP, or local support services |  |  |

## Appendix 5. Topic guides

**INTRODUCTION**

- Introduce researcher
- Introduce project
- Include here about how we are sorry about what happened to family taking part. Let participants know that we will not expect them to share the details of the event that led to their adverse outcome, and that it is up to them if they would like to do so during the interview, in a pre-interview meeting or in advance in writing. Emphasise that we are happy to listen if they would like to share
- Outline what will discuss in the interview
- Emphasise that there are no right or wrong answers and taking part is completely voluntary, and that they are free to withdraw at any time
- Explain that participants can pause or stop the interview or skip questions at any time
- Say that the interview will be audio recorded and professionally transcribed, but findings will be fully anonymised (names, places) – check okay to record
- Check if any questions?
- Thank for providing consent ahead of interview, or take verbal consent

**Questions for those who have received support from the MNISA**

| **Topic** | **Main question** | **Follow up question (prompts)** |
| --- | --- | --- |
| **Introduction***(note this question may be covered at the start of an interview or in a separate pre-interview conversation)* | If you feel comfortable to, would you like to tell us about your baby(ies)/family/wife or partner (depending on adverse outcome experienced)? | - Would you like to share your child’s/family member’s name and use it throughout the interview? - Is there anything else you would like to share? |
| **Break** | ***Option for a break*** | ***Only record from this point OR give the option to only record from this point** |
| **Relationship with MNISA** | How would you describe your relationship with your Maternity and Neonatal Independent Senior Advocate? | - How did you develop this relationship/rapport? - How has this helped or got in the way of you receiving the support needed? - What is the quality and frequency of communication and updates? - Transparency with you – what has been done/when and with what outcome? |
| **Support received** | *We know that you have received support from XX who is a Maternity and Neonatal Independent Senior Advocate. These questions will focus on the support that you have received from her/him so far.*   1. Please could you describe what you thought they could help you with? And why they were recommended to you? | - How did you find out about the MNISA? - How were you referred/how did you access the role? - What is your understanding of the role and service? - Did the hospital mention any processes they would use to look into what happened? - From your point of view, why do you think the role was developed?/why it is needed? |
| **Experience of support received** | 1. How have you found the support received from your Maternity and Neonatal Independent Senior Advocate? | - What was your understanding of what the MNISA would be helping you with? (e.g., help to engage with other departments or agencies, helped to engage with complaints team, helped with accessing records) - From your point of view, how is the role different/similar to other services you may have received? (health care workers (HCWs), investigation staff, volunteers, bereavement nurse etc) - What information did you receive about the MNISA? - What format was the information in (leaflet, email, telephone, other) - How would you have preferred it? - How long have you interacted with your MNISA so far? - Did you need support from the outset or was there sufficient information from other professionals? - When did you feel you needed MNISA support? - How many appointments? - Mode of appointment? - Type of things they help with - At what stage is the support most useful? - Is there anything you’ve not had help with yet, that you think they could support you with? - What did you like? - What don’t you like? What would you improve? - Does the support received match your expectations of what you would like to receive? - If yes, how? (please give examples) - If no, why? (please give examples) - How may have things been different for you without this support? - Does the MNISA have sufficient knowledge about processes involved in your case? |
|  | 1. What helped and got in the way of you initially accessing support with the Maternity and Neonatal Independent Senior advocate? | - What helped? - What got in the way? |
|  | 1. What has helped and got in the way of you engaging with the Maternity and Neonatal Independent Senior Advocate? | - What helped? - What got in the way? |
| **Meeting intended outcomes** | *The next few questions, we will ask you about how you think the Maternity and Neonatal Independent Senior Advocate is meeting the outcomes that NHS England hoped for when setting up this role*   1. NHSE wanted MNISAs to support women and families to feel heard and listened to. Was that something that you wanted from the MNISA? Do you think the support you have received from the MNISA has or may achieve this? | - Please give examples as to why - Are you confident that issues which have been raised have been listened to but also fully understood by the MNISA? |
|  | 1. NHSE wanted this role to support women and families to be confident that their concerns have been listened to and acted upon. Do you think that the support you have received from the MNISA has or may achieve this? | - Please give examples as to why - Are you satisfied that your concerns have now been escalated and action is being/will be taken by your MNISA? |
|  | 1. Are you aware of any changes the Trust (local services), wider, national or healthcare system (more widely across location, different pathways etc) has made as a result of your incident/baby’s death? | - If so, do you think the MNISA directly helped to enable this change? And how? - If no, what change would you have liked to have seen? And why? |
|  | 1. *Where applicable:*Did the MNISA ensure that all safety concerns have been resolved and you received all information needed before closing your case? | - Please give examples |
| **Views on the role more generally** | We are interested in learning about whether you feel that the MNISA position is set up in the best way to support you:   1. From your experience, what are your views on: 2. The amount of time MNISAs have available 3. The independence/impartiality of the MNISAs 4. The seniority of the MNISAs 5. The background and experience of your MNISA (are you aware of that?) 6. Where the MNISA is employed 7. EDI – accessibility of the role 8. Who the MNISA is employed by (are you aware of that?) | 1. Do they have enough time to support you? 2. Are they independent/impartial enough to support you? What does independence mean to you?  (operational guidance: MNISAs being separate from the trusts/those responsible, transparency around governance, reporting and escalation) 3. Are they senior enough to support you? 4. Do they have the right skills/experience to support you (clinical vs non-clinical)? Do these skills enable them to achieve the duality of the role? 5. Are they employed in the right part of the healthcare system? 6. Did they have sufficient system knowledge to support you?     For each, please tell us a bit about why and whether there is anything that you would change |
| **Reflections on future practice** | 1. What are your thoughts on how MNISAs could be used in future? | - Should the role continue to be used, or should it be discontinued? - Would you recommend any adaptations to the role? - Scope of the role? - Expectations for support received? - Have a wider influence on the system? - What needs to change to make it effective? |
| **Wrap up** | 1. Is there anything else you’d like to say that we haven’t spoken about so far? |  |
| **Demographic characteristics** | - Gender/sex - Age - Area of the UK live in (i.e. which ICB live in or receive care in) - Ethnicity - Relationship status - Sexuality/sexual orientation - Level of education - Postcode - Gender identity - Disability (or health problem) - Category of adverse outcome experience – *[options below are from NHS operational guidance for the service]* - Stillbirth (after 24 weeks) - Neonatal death - Maternal death - Unexpected or unplanned hysterectomy (within 6 weeks of birth) - Women admitted to critical/intensive care - Brain injury that has been diagnosed or suspected, including hypoxic-ischaemic encephalopathy (HIE) - Time since adverse outcome |  |

**DEBRIEF**

- Ask participants if they are happy for us to follow up with them via email (or another preferred method) after the interview to check that they are okay.
- Check if they have someone to talk to following the interview if needed. If they do not have someone to talk to, we will signpost them to the Sands helpline, if needed. Our Sands team will brief helpline staff about scheduled research activity.
- Provide participants with a debrief form following each interview, which signposts to key charities and local sources of support.

**Questions for those who declined support from a MNISA -**include here about how we are sorry about what happened (Sands feedback). Explain about recording and when they would like to start the recording.

**INTRODUCTION**

- Introduce researcher
- Introduce project
- Include here about how we are sorry about what happened to family taking part. Let participants know that we will not expect them to share the details of the event that led to their adverse outcome, and that it is up to them if they would like to do so during the interview, in a pre-interview meeting or in advance in writing. Emphasise that we are happy to listen if they would like to share
- Outline what will discuss in the interview
- Emphasise that there are no right or wrong answers and taking part is completely voluntary, and that they are free to withdraw at any time
- Explain that participants can pause or stop the interview or skip questions at any time
- Say that the interview will be audio recorded and professionally transcribed, but findings will be fully anonymised (names, places) – check okay to record
- Check if any questions?
- Thank for providing consent ahead of interview, or take verbal consent

| **Topic** | **Main question** | **Follow up question (prompts)** |
| --- | --- | --- |
| **Introduction** | If you feel comfortable to, would you like to tell us about your baby(ies)/family/wife or partner (depending on adverse outcome experienced)? | - Would you like to share your child’s/family member’s name and use it throughout the interview? - Is there anything else you would like to share? |
| **Break** | ***Option for a break*** | ***Only record from this point OR give the option to only record from this point** |
| **Support received** | *In your local area, there is a role called ‘Maternity and Neonatal Independent Senior advocates’ who can offer support to families who have experienced specific adverse outcomes. Their role is to support women and families to feel heard and listened to, to help them to ensure that their concerns have been acted on, and to create change or learning at a Trust and organisational level.*     1. Are you aware that this service is available (or was available at the time of your incident)? | - How did you find out about the MNISA support? - What information did you receive about them? |
| **Decision not to access service** | 1. Could you tell us a little bit about why you chose not to access support from the MNISA? | - Was anything off putting? What would have better met your needs? Did you need it? - Were you satisfied with the information and actions taken by the treating team, or support already available through different avenues, by PALS, MNSI  or volunteers or bereavement nurse for example? - What did you dislike about the role? - Did you feel the service would be useful/accessible to all parents/families? |
|  | 1. Are there any other types of support that you would have wanted, or would like moving forward? | - [Trust level, ICB level, charity level etc] - Who would you want to provide that support? (independence/seniority/skills and experience/time/where employed? |
| **NHSE intended outcomes** | *The next few questions, we will ask you about your thoughts on whether/how the Maternity and Neonatal Independent Senior Advocate might be able to meet the outcomes that NHS England hoped for when setting up this role*   1. NHSE wanted MNISAs to support women and families to feel heard and listened to, how much do you think that the support from a MNISA could achieve this? | - Please give examples as to why |
|  | 1. NHSE wanted this role to support women and families to feel that their concerns have been acted on, how much do you think that support from a MNISA could achieve this? | - Please give examples as to why |
|  | 1. How much do you think MNISAs would be able to support with change or learnings at Trust (local services) or healthcare system level (more widely across location, different pathways etc)? | - Please give examples as to why |
|  | 1. Could you tell us a little bit about any steps you have had to take to enact change (either on your own or collaboratively with others?) (if relevant) | - Who did you need to contact? - What happened? - What changes were they aware of that were made? - Would a MNISA have helped with this from your perspective? If so how? |
| **Reflections on future practice** | 1. What support structures do you think might be helpful in the future for supporting families who have been through adverse outcomes? | 1. Continuation of MNISA roles? 2. Other roles? 3. Other structural factors?  - Trust level/ICB level/charity level? - Why? |
| **Wrap up** | 1. Is there anything else you’d like to say that we haven’t spoken about so far? |  |
| **Demographic characteristics** | - Gender/sex - Age - Area of the UK live in (i.e. which ICB area live in or receive care in) - Ethnicity - Relationship status - Sexuality/sexual orientation - Level of education - Postcode - Gender identity - Disability (or health problem) - Category of adverse outcome experience – *[options below are from NHS operational guidance for the service]* - Stillbirth (after 24 weeks) - Neonatal death - Maternal death - Unexpected or unplanned hysterectomy (within 6 weeks of birth) - Women admitted to critical/intensive care - Brain injury that has been diagnosed or suspected, including hypoxic-ischaemic encephalopathy (HIE) - Time since adverse outcome |  |

**DEBRIEF**

- Ask participants if they are happy for us to follow up with them via email (or another preferred method) after the interview to check that they are okay.
- Check if they have someone to talk to following the interview if needed. If they do not have someone to talk to, we will signpost them to the Sands helpline, if needed. Our Sands team will brief helpline staff about scheduled research activity.
- Provide participants with a debrief form following each interview, which signposts to key charities and local sources of support.

## Appendix 6. National debrief sheet

**Maternity & Neonatal Independent Senior Advocates (MNISAs): A Rapid Mixed Methods Evaluation**

Thank you for taking part, we are grateful for your participation. Your views are important in evaluating the MNISA role.

**What happens next?**

We will ensure that any information which identifies you is removed from the transcript. Please do let us know if you would like a copy of your transcribed interview in due course. We are happy to share a redacted copy of your transcript with you if would like to see it.  Please be assured, the audio recording will be deleted when it has been transcribed and one of our researchers has checked the transcription is accurate.

**What will happen to the findings?**

In June 2025, study findings will be used to evaluate the impact of the MNISA role and inform how the role may be implemented moving forward. Findings will be shared in a variety of ways including reports, academic publications, presentations and plain English summaries, to a variety of audiences. Once published, the findings will be accessible on the RSET (Rapid Service Evaluation Team) website.

**Where do I go if I have any questions about the study and my involvement?**

If you would like to be added to a mailing list, where you can be provided with updates and published findings when they are available, please email us: [mnisastudy@ucl.ac.uk](mailto:mnisastudy@ucl.ac.uk)

If you have any questions about the project or your participation, please email us: [mnisastudy@ucl.ac.uk](mailto:mnisastudy@ucl.ac.uk)

**Where do I go if I feel I need more support?**

We have provided a list of support services and/or organisations below.

*Key support organisations/charities:*

- **SANDS:** If you have been affected by pregnancy loss or the death of a baby you can speak in confidence to the Sands Bereavement Support team by calling the Sands helpline, open Mon-Fri 10am-3pm and Tue-Thur 6-9pm on 0808 164 3332 or emailing [helpline@sands.org.uk](mailto:helpline@sands.org.uk)
  - More details about the support that Sands offer can be found here: [How we offer support | Sands - Saving babies' lives. Supporting bereaved families.](https://www.sands.org.uk/support-you/how-we-offer-support)
  - Contact details for the Sands Bereavement Support team (Sands helpline, open Mon-Fri 10am-3pm and Tue-Thur 6-9pm): telephone: 0808 164 3332; email: [helpline@sands.org.uk](mailto:helpline@sands.org.uk)
- **Cruse Bereavement Support:** UK leading bereavement charity which supports everyone who is grieving. They provide a helpline and also provide individual support.
  - More details can be found here: [Cruse Bereavement Support](https://www.cruse.org.uk/about/contact-us/)
  - Telephone: 0808 808 1677
- **Petals:** A charity which provides support for parents and families following baby loss. They provide specialist counselling for parents affected by: miscarriage, poor pregnancy diagnosis, termination for medical reasons, stillbirth, neonatal loss, antenatal anxiety following a previous loss/losses.
  - More details can be found here: [Petals, The baby loss counselling charity - Petals Charity](https://www.petalscharity.org/)
  - Email: [contact@petalscharity.org](mailto:contact@petalscharity.org)
- **Tommy’s:** A charity which works to make birth safe for everyone. They fund research to identify why pregnancy goes wrong and how to prevent complications, provide midwife-led during and after pregnancy and support people who have lost babies.
  - More details can be found here: [Saving babies' lives - Charity for Babies | Tommy's (tommys.org)](https://www.tommys.org/?gad_source=1&gclid=EAIaIQobChMIqIb4g-26hwMV65JQBh0EPwH-EAAYASAAEgId0vD_BwE)
  - Email: [mailbox@tommys.org](mailto:mailbox@tommys.org)
- **PEEPS:** UK charity dedicated to supporting people affected by hypoxic-ischaemic encephalopathy (HIE). They provide support to parents, families and friends of those affected by HIE and aim to raise awareness of HIE.
  - More details can be found here: [Peeps HIE Charity | HIE Awareness & Support (peeps-hie.org)](https://www.peeps-hie.org/)
  - Email: [info@peeps-hie.org](mailto:info@peeps-hie.org)
- **Action Against Medical Accidents (AvMA)**: Independent charity for patient safety and justice who support people following a medical incident.
  - More details can be found here: [AvMA - Help and advice](https://www.avma.org.uk/help-advice/)
  - Helpline 0345 123 2352 (Monday-Friday 10am-3.30pm - 03 calls cost no more than calls to geographic numbers (01 or 02) and must be included in inclusive minutes or there can be a cost per minute)
- **Birth Trauma Association:** UK charity which provides support for women and families who have experienced a traumatic birth. They offer peer support services, influence policy and contribute to research and campaign to raise awareness to help improve maternal safety.
  - More details can be found here: [The Birth Trauma Association](https://www.birthtraumaassociation.org/)
  - Email: [support@birthtraumaassociation.org.uk](mailto:support@birthtraumaassociation.org.uk)

*Additional avenues of support:*

- Your local GP

## Appendix 7. Additional quotes table

| **Section/page (in findings section)** | **Topic/theme** | **Additional illustrative quotes** |
| --- | --- | --- |
| ***Experiences of families who did, and families who did not, receive support from MNISAs*** | | |
| Page 13 | Factors influencing access to the service *(awareness of the service)* | *“At first, I was not 100% sure about her. Because I have so many problems and I [worried this would be] yet another problem I'm not going to resolve, you know? But then she came to my house and said, ‘I'm going to help you’. I talked to her, and I was still a bit 50/50, you know? But once she started to be involved in our case, the case started moving.”* (Father whose baby had a brain injury, INT16)  *“Initially, I wasn’t fully aware of the significance of their role or the range of services they offered. This lack of understanding delayed my decision to contact them”* (Family whose baby died in the neonatal period, INT12)  *“I just remember being like, ‘oh my god, someone’s like asking if they can help me. And it was just like, oh my god, someone’s going to come and listen to my story [cries] and be able to help me function”* (Mother whose baby had a brain injury, INT2)  *“We received a pamphlet from the midwife who supported us during the bereavement. At the time, I didn’t fully understand the importance of her role. Occasionally, I’ve wondered what might have happened if I had overlooked the pamphlet and ignored it”* (Family whose baby died in the neonatal period, INT11)  *“I was trying to dig into the ICB and how did the ICB not know about the SIs and the CYC, even trying to link it all and there was just this webpage linked to the maternity advocate. I then read about it and was like, how, having had three babies, not just one, how did I not know this person existed? How has nobody, I only found out towards the end of last year, how has nobody referred me to this person for support? That’s kind of when this came up, I was like hang on a minute, this makes perfect sense. I did not know about this, they don’t advocate this”* (Mother who was admitted to critical/intensive care and did not access the service, INT32) |
|  | Factors influencing access to the service *(distrust in the hospital and timing of the offer)* | *“[The hospital] didn’t really explain it to us. And now I think about it, why would they? Why would they want an independent, experienced NHS operator opposite them in serious meetings? I’m just speculating as to why that might be. Why we didn’t know”* (Family whose baby was stillborn, INT21)  *“Just to give you some context, in the letter, they wrote, ‘I'm sure it's a disappointment that you won't be entitled to compensation’. What? I don't need your money! With the greatest of respect, I don't even want your money. I haven't asked for this. I'm not an ambulance chaser. The NHS - It's actually questioned my entire like, do I still want to work for an organisation that does business like this? That's how deep seated it is in me. Every time you get a communication from someone else in the NHS that says something like that, it just makes me even more…It's like there's no sincerity in it. I'm not asking for money. I want answers and I don't believe that what you're giving me there are the answers. When you can't even get our address right when we've sent it months before in writing. It's just those little things that sort of wind up making me think, well, if you can't even do something like that right, how do I know that the investigation’s been done right? How do I know that's been done right?”* (Father whose baby had a brain injury, INT28)  *“Had I found out about the MNISA through different means, I think I would’ve been more trusting of it. But because the hospital recommended her themselves, I was really reluctant to accept that support because I just felt like everything they were doing was biased at that point. I think initially, they thought that me using the MNISA was probably working in their favour. However, I think they have a different opinion now because [...] I have found lots of information through her. I don’t think they understood like how much more.”* (Mother whose baby died in the neonatal period, INT23)  *“We just didn’t know what support the role could offer. Those early days are filled with shock, denial, feeling lost, despite being handled information. It was the week after when our bereavement midwife mentioned it to us again and that is when we could finally sit and listen properly”* (Mother whose baby had a brain injury and died in the neonatal period, INT26)  *“You’re trying to process the grief and loss and all of the things around – you’d prepared to have a life with a baby and then you’re just in a silent house”* (Mother who was admitted to intensive care and whose baby was stillborn, INT30)  *“The emotional overwhelm of the bereavement process itself made it difficult to focus on seeking help or taking the steps to register for support right away”* (Family whose baby died in the neonatal period, INT12) |
| Page 14-15 | Type of support received *(navigating reviews, investigations and systemic processes)* | “*It’s really sad...I’m on the WhatsApp community of stillbirth mums and they’re all going through the PMRT process, and they have no clue what the process is. For some of them the process has already happened, they didn’t even know it was happening they didn’t get the chance to submit questions, and they’ve had a totally opposite experience from me. And now a year later, they’re having to – because that’s the thing, it’s only now I’ve got the strength to really delve in and ask the questions.”* (Mother whose baby was stillborn, INT19)  *“I am living everyone’s worst nightmare and having somebody there that can actually help guide in that situation, it’s like essential....[...] You know on those old maps that they used to draw, ‘There’d be Dragons!’. You don’t know what to do or what was there. I was in those waters. Having somebody that had some idea where there might be some land, it’s very difficult to describe what it does inside your head.”* (Father whose partner died giving birth, INT8)  *“Now, without [our MNISA], and without me being able to bounce these conversations off her and walk through it and answer some of those questions to allow me to pull the next thread, I don't think we'd have got this far. Because I've been able to say, ‘But why does it just say calcium?’ She’d say. ‘I don't know’. Those little bits that we've walked through together to build up a picture, but at no point has she necessarily told us to do anything. But it's been helpful to have someone there who I can, who I can have those conversations with, and who knows enough about it to have a conversation where you can get further down that road. I don't know if we'd have done it without [our MNISA] there because my brother wouldn't have probably been pushing very hard for me to do it because I'm very busy. So because [our MNISA] has put in that hard work. I've put in mine. That's been a bit of a driving force.”* (Family member of mother who died giving birth, INT9)  *“[The challenge is] going back and forward with the different systems and the hospital. It’s quite, it’s emotionally really difficult to deal with when you’re at that point,. you know, when you have to start doing other things in life, like I had to be back at work after maternity leave. There was too much to keep doing all of that, just driving it all myself, and not knowing how it worked. It would have been really helpful to have someone to ask who knew the systems”* (Mother whose baby had a brain injury, INT2)  *“The support that [our MNISA] provided was very much welcomed in a time where we just felt like we weren't getting anywhere. And to be perfectly honest, we just wanted the process over and done with. It's like I said, we didn't ask for any of this. We didn't ask for the HSIB investigation. I didn't ask for it to be referred to NHS Resolution. I didn't ask for the language that came through in the communications [from the hospital]. I mean, I dread to think what an uneducated family would have done with it”* (Father whose baby had a brain injury, INT28)  *“I was just left, stuck with a lot of questions and just needed answers really. For the most part, it kind of feels like quite a lonely journey.”* (Mother whose baby died in the neonatal period and had not accessed the service, INT34) |
| Page 15-16 | Type of support received *(comprehension of care)* | *“Our mother tongue is Tamil, and we felt that having a Tamil speaking psychiatrist would help my wife speak more freely and from her heart. We conveyed this information to our MNISA and the midwife from the hospital. Unfortunately, the support we've received from the hospital was non-existent. However, our MNISA went above and beyond by finding a Tamil speaking psychiatrist nearby and even arranged for the Trust to cover the cost. This was tremendous help during such a difficult time.”* (Family whose baby died in the neonatal period, INT12)  *“You need somebody that speaks the language, so to speak. To be able to put it into layman’s terms. It’s almost like having a solicitor. The reason you have a solicitor is because in court they speak legalese, so you need a translator there. The advocates are the translator that’s there to help you get through the shi**iest thing ever.”* (Father whose partner died giving birth, INT8)  *“We just felt stuck in a lot of ‘what if’. We were just left. Yeah possibly having somebody who could have advocated for us probably would have helped a lot”* (Mother whose baby was stillborn and did not access the service, INT33) |
| Page 16 | Type of support received *(a continuous point of contact)* | *“I’m still pushing for answers to questions. There are times when I’ve wanted to give up because it just seems too hard. [My MNISA] just said, you know ‘let’s finish now because it’s emotionally overwhelming. But don’t lose sight of the questions that you still want answers to because that’s what keeps you up at night. And she’s absolutely right, so she’s really helped just with the continuity when you would otherwise feel like you need to – You just need to give up”* (Mother whose baby was stillborn, INT31)  *“[Our MNISA] was my person to talk to. She’s just been there all the time, no matter what I need. It’s been really, really helpful to have her”* (Mother whose baby died in the neonatal period, INT1)  *“She is there all the time with us, any question, any small problem, she is there*” (Family whose baby died in the neonatal period, INT29) |
| Page 17 | Factors influencing ongoing engagement with the service *(MNISA experience, knowledge and approach)* | *“They’re not allowed to tell her ‘no’, sort of thing. They have to listen to her. With families, I guess it’s easy to kind of just gaslight them and shut them down and tell them ‘no, you can’t do that’. But I suppose it’s different when there’s a person who knows the rules”* (Family whose baby had a brain injury and died in the neonatal period, INT18)  *“It was less about having an expert sat there but more somebody who was empathetic and wanting to support this journey, which is so rocky”* (Mother whose baby was stillborn, INT19)  *“It [MNISA support] was always on our terms. It was like when we had to go into the hospital, “actually are you okay to go back into that place? Do you want to sit somewhere else, or do you want to be somewhere completely different?” It was just – yeah, she made sure we were always comfortable”* (Joint interview with family whose baby was stillborn, INT21)  *“It’s like rather than it being her job from our perception, it’s more like a lifestyle that she’s adapted. Like, she just lives that role rather than her getting paid to do it”* (Family whose baby died in the neonatal period, INT22)  *“Health care and something as serious as working with children and babies and stuff, you’ve got to be all in. You’ve got to be fully committed and all in and go the extra mile. Because if you slack and you do half a job then it could be tragic consequences as we’ve seen”* (Father whose baby died in the neonatal period, INT27)  *“[What would have been helpful is] Someone that really understands the different systems and how they fit together. And then also someone that understands the impacts on the parent or the patient who’s gone through whatever the situation is. Well, just having a bit of context for what that would feel like for the person that’s going through it because being compassionate is very important because you’re constantly facing this really medical and impersonal language and all these impersonal systems. So that human touch is missing and it’s quite brutal”* (Mother whose baby died in the neonatal period and who did not access the MNISA service, INT34)  *“I think it’s a bit more important to understand the system to be supportive in their advocacy, definitely, because this is the most detail aspect. For the more medical context, they’d probably know who to ask to get more information.”* (Mother whose baby died in the neonatal period and who did not access the MNISA service, INT34)  *“I definitely think her background in neonatology helped us. We're both medical doctors, but we are adult doctors. We obviously have some knowledge of neonatology and obstetrics, but not enough. So she was kind of, again, she acted as a bridge to help us understand what was going on. Because we are healthcare physicians, clinicians and parents. She helped us stay in separate roles; she helped us stay parents rather than being healthcare professionals.”* (Family whose baby died in the neonatal period, INT29) |
| Page 17-18 | Factors influencing ongoing engagement with the service *(independence from the hospital)* | *“She was very professional. She’s there for us, but I also know that there are boundaries and things. She’s good with the hospital as well, she sends them emails, I think it’s just the way she words things as well. She always seemed to have good relations with the doctors and whoever we’re meeting with, they knew her. But yeah, then she would be helping us as well. She balanced it well”* (Family whose baby had a suspected brain injury and died in the neonatal period, INT18)  *“Even though her role is funded by the NHSE, she doesn’t work for the hospital that’s under investigation, which is a massive thing for us. Because we find it very hard to trust people right now, particularly the hospital that are under investigation and everyone that we were speaking to was employed by the hospital. So we just felt on our own really, before she came along”* (Family whose baby died in the neonatal period, INT21)  *“There’s so much corruption, where practice is questionable that it has to be an external expert”* (Mother who was admitted to the critical/intensive care unit and did not access the MNISA service, INT32)  *“Before our MNISA, the hospital were never hostile, they always tried to explain things, but we already felt that there were two teams in the room. No one is fooling us. But it gives us reassurance that actually we have a person who knows. Like [our MNISA] has done numerous PMRT's back in her day. So she knows the process and she helped me understand the PMRT. And where the Trust actually again not deliberately, but like, if you go through everything yourself, but you still then question, is the Trust being very, very honest or not? So like we can openly talk to a person who is actually working within the system. And is actually on our side. And we have seen that we can ask her anything. We can literally accuse the Trust of anything. And she would give us a very honest answer, not being too biassed on either side. So we find her very open and quite accurate with things. It's been a blessing”* (Family whose baby died in the neonatal period, INT29) |
| ***Perceived impact of MNISA support*** | | |
| Page 18-19 | Psychological impact for families *(alleviating the emotional overwhelm families experienced)* | *“There's times that I felt a bit of guilt because I felt the most important thing should be getting answers, and preventing things that we think went wrong. But I've been pulled in other directions, and I think having [our MNISA] sort of takes a lot of that guilt away. Instead of me having to sit down for two hours at the end of the day and write a letter or try and condense things, I just send her an e-mail and she'll say, ‘oh, I'll do that’. That's the big thing, because I know it's being done properly, it takes that pressure off of me. Because then it means I've got time to spend with [my toddler]. Or just decompressing instead of having to worry about doing right by the girls.”* (Father of twins who died in the neonatal period, INT10)  *“I can't imagine where we would be. I don't think we'd have been able to function like we have been since we've had her, you know? Every day was just another stress. We were stressing over e-mails. Again, still burning questions which - We've just lost a child - like the last thing we need is to have that stress. More worries on top of everything else. And again, when you've got other children, and life doesn't stop, the world keeps spinning, you've got to carry on. But the fact that she's been able to take that stress away, we've probably just been able to be a bit more present for our children. Just get up in the morning and feel a lighter load. Yeah, I can’t explain what she's done for us in just a matter of few weeks.”* (Family whose baby died in the neonatal period, INT22)  *“As [her] Mum I wanted to do everything, but I was dealing with grief as well. It’s knowing where to go, what support. She [our MNISA] supported us in us knowing that she’s there for [my daughter] and managing to go through this journey with her in terms of looking at the complaints, asking the questions that we don’t know the answers to.”* (Grandmother whose granddaughter was stillborn, INT19)  *“[My husband] said it like a million times. I'm just so pleased you've got somebody to have that conversation with. You know, I'm so pleased you're able to get that off your chest today. See, you were right all along, and we don't need to worry. Yeah. Yeah. It's just it's a role that he can't fulfil. But that he's grateful, can be fulfilled”* (Mother whose baby was stillborn, INT31)  *“Because if you look, we are both [healthcare professionals], we both are an important cog, a small, but important cog in society, we both pay our taxes, productive component of society. Now reverse it and say that we both become severely mentally impacted by all of this, we couldn’t work, now we are a burden. So, I look at it like this, [our MNISA] is helping parents get back on track”* (Family whose baby died in the neonatal period, INT29)  *“Taking the stress, taking as much stress away from us as she could. You see what I mean? Because the stress is always in the background, of ‘when is this going to end?’ kind of thing. But doing all the legwork, doing all the chasing, using her contacts with people further up in the organisation to say this isn't good enough, what are you going to do about it? Things I would have physically, even if I wanted to.”* (Father whose baby had a brain injury, INT28) |
| Page 19 | Enabling family voices to be heard *(feeling listened to and validated)* | *“[I was] just so grateful that [our MNISA] was just there and just listened. She just let me talk and I felt really safe with her”* (Mother whose baby had a brain injury, INT2)  *“She just listened really well”* (Mother whose baby had a brain injury, INT25)  *“She understood our concerns and was able to clearly display how she'd understood it, because she then emailed us back, I think it was either in the evening or next day with the long lists of all our concerns. And she had actively listened.”* (Father of babies who died in the neonatal period, INT10)  *“During such an emotionally overwhelming time, it was crucial for us to have someone who could not only listen to our concerns but also understand the depth of our feelings and help us to navigate the situation with compassion. The support we received from [our MNISA] fully achieved this. They created a safe and empathetic space where we could express our concerns without fear of judgment. They took the time to listen attentively, validated our emotions and ensured that we felt understood throughout the process”* (Family whose baby died in the neonatal period, INT12) |
| Page 19-20 | Enabling family voices to be heard *(ensuring family concerns are acted upon)* | *“Our MNISA went through all the drafts and everything with us and we'd spend hours going through each draft report— pulling it apart. Making notes to take back to the hospital. Our MNISA was fundamental in that because it was a lot of information. The report was about our son. It was not about our MNISA’s son. We brought the full emotional attachment to those reports whereas our MNISA could stay calm take a step back and see the report for what it really said.”* (Father whose baby died in the neonatal period, INT27)  *“She was really good at interpreting what I was saying. So, if I was saying I don't understand why the ambulance did this, she'd be like, well, why don't you ask that question? She'd be like noting all my questions down so that it just took away that...If someone had asked me to sit down and write a letter and ask the questions I wanted answers to, I wouldn't have been able to. And didn't have the capacity to do it.”* (Mother whose baby was stillborn, INT19)  *“[Speaking about her experiences to her daughter]. You had the questions, you were verbalising the questions, but your MNISA put them into words. If someone had said, ‘Sit down and write a letter and ask the questions.’ You wouldn’t have been able to. You didn’t have the capacity. Our MNISA literally collated all our questions, and it was in those moments – It certainly impacted our ability to grieve as a family and support [my daughter] as parents - not as go-betweens between the hospitals.”* (Grandmother whose granddaughter was stillborn, INT19)  *“How can we then feedback what happened with [our baby] and what went wrong to the right person, because we’re feeding everything back to the hospital. [Our MNISA’s] the one that made the connection so that the ambulance service were actually involved. The investigation team, they do so much but it doesn’t seem to pull together at the end. I found that [our MNISA] was the one that pulled it all together and made sure actually everything was fed back to the right people that will help moving forward”* (Mother whose baby died in the neonatal period, INT20)  *“I think that me going to the meeting with [our MNISA] gave the meeting authority. So, when I went into the meeting and they were introducing themselves and they had a Dictaphone with them as well. I said, ‘This is the advocate that I'm working with. I'm [their name] and I'm [their baby’s] mum’. And she then introduced herself. It kind of just gave it that formality and that authority. This is someone I'm working with to make sure things are done properly and things are followed up”* (Mother whose baby had a brain injury, INT2)  *“Without a doubt. Obviously, I'm really familiar with reading reports with clinical jargon, with arguing with an outcome, because I fight for people's rights all day, every day. But when you've got to do it for yourself, it's too emotional. You can't actually separate the black and the white to put your argument forward. That's what [our MNISA] did for me. She said, ‘You talk and I'll write. You just breathe’. And I was just like, Oh my God, I haven't got to do all that?! It was a massive weight off my chest. I felt like I was going to have to write a dissertation type response to MNSI when I was completely incapable of anything academic really”* (Mother whose baby was stillborn, INT31)  *“[After we got our PMRT report] I sort of read it and thought, oh, that’s amazing that they’ve put that in place and [our MNISA] said to us, “Do you actually know that that means?” and I said “Well, not really but it’s good that it’s in place.” And our MNISA said “Well I can go and find out what they’ve actually put in place and find out if they’re actually doing it. It’s easy to say on paper, “Yes, we’re going to do this and we’re going to do that. But our MNISA can now go follow it up and say, “Right, are you actually doing it?” It’s nice to know it’s actually going to be implemented”* (Family whose baby died in the neonatal period, INT21) |
| Page 20-21 | Examples of family-led changes | *“I suppose what I’m saying is I would have no problem [making change with MNISA support]. I just haven’t broached it yet because one thing at a time so to speak. But [our MNISA] has offered, she did make that clear, that if there’s anything that she needed to do in terms of the Trust, anything that I wanted following up, chasing up, she was happy to do so. I just haven’t had the chops to do it, for want of a better word, at the minute”* (Father whose baby had a brain injury, INT28)  *“She can give the family a voice. But making the system change isn’t up to her. She can’t facilitate the change. She can only communicate advice for changes. Then the people on the receiving end of that advice have obviously got all of their cultural oppression from working in where they work. But then how far that actually gets I think would be questionable. Because what she says and what she brings to the table it’s just really comprehensive. It’s how it’s received at the other end that’s the battle”* (Mother whose baby was stillborn, INT31)  *“I am a nurse. At [our hospital] I think there is a reluctance to change. People don’t like outsiders coming in and telling them what to do, and that’s across all departments. That is not the norm that I’ve ever experienced. Change should be able to happen. If you think of what the NMC and GMC stand for, although they’ve got their issues, it should be that we are evidence-based. We are looking at research. We are educating ourselves. We are constantly striving for improvements, better healthcare. Everything is re-evaluating, self-reflection, global reforms, global reflection. They [the hospital] don’t do that.”* (Mother who was admitted to critical/ intensive care and did not access the MNISA service, INT32)  *“We’ve already been told, just from one first, you know, initial meeting with the lead investigator, that there are going to be learnings taken from what’s happened. Probably a lot of learnings already and we’re right at the beginning of the process. I think because [our MNISA] - unfortunately - only been in our lives for a couple of weeks. The information we’ve got regarding how long the investigation is going on for is slim. But she’s already made a massive difference in a short period of time”* (Family whose baby died in the neonatal period, INT22) |
| Page 28 | Future of the service | *“I absolutely think it should be continued, and my rationale behind that is for all the things that we’ve talked about: the support, allowing families to feel heard in a system that sometimes feels that they don’t want you to be heard, like to brush things under the carpet”* (Father whose baby had a brain injury, INT28)  *“It should be utilised in the future. During a time when you don’t know where to go, it’s so helpful to have someone. Someone to speak to, to take guidance from, talk things through. Feels like you have someone fighting your corner”* (Family whose baby died in the neonatal period, INT14)  *“Please don’t discontinue this role. We’d be devastated if it got discontinued. I can’t see any negative points of her role; there’s nothing negative at all”* (Family whose baby died in the neonatal period, INT22)  *“The role absolutely should continue to be offered. We simply would have been lost without it and not achieved anything. We have learnt so much and received many answers that we would not have got without the service and are continuing to achieve more just by having someone professional helping in your corner. Having an advocate helps in holding people to account and I think without [MNISAs] families like us would have just been ignored and had no further guidance on where to go”* (Family whose baby died in the neonatal period, INT12)  *“I strongly believe that the MNISA role should continue to be offered to families and in fact, I think it should be more widely promoted as a vital support service”* (Family whose baby died in the neonatal period, INT12)  *“I think [the MNISA service] has to be there. What I’ve been through isn’t nearly anything as what some other people have been through. And like I said, I would not have got through, or I wouldn’t be in the place I am now without that role being in place”* (Mother whose baby had a brain injury, INT2)  *“It scares be the idea of it not [continuing] to be honest. I don’t understand how that [MNISA support] could disadvantage a family at all”* (Mother whose baby was stillborn, INT19)  *“I do feel though that there's always the element of just trying to keep people at arm’s length and do what needs to be done as opposed to which should be done. [Our MNISA] was the first time that didn't feel the case. And I think it would be worthwhile if it could be ramped up to support more families”* (Father whose baby had a brain injury, INT28) |
